# A programmable topological photonic chip

**DOI:** 10.1038/s41563-024-01904-1

**Published:** 2024-05-22

**Authors:** Tianxiang Dai, Anqi Ma, Jun Mao, Yutian Ao, Xinyu Jia, Yun Zheng, Chonghao Zhai, Yan Yang, Zhihua Li, Bo Tang, Jun Luo, Baile Zhang, Xiaoyong Hu, Qihuang Gong, Jianwei Wang

**Affiliations:** 1 State Key Laboratory for Mesoscopic Physics, School of Physics, Peking University, Beijing, China; 2 Division of Physics and Applied Physics, School of Physical and Mathematical Sciences, Nanyang Technological University, Singapore, Singapore; 3 Centre for Disruptive Photonic Technologies, The Photonics Institute, Nanyang Technological University, Singapore, Singapore; 4 Institute of Microelectronics, Chinese Academy of Sciences, Beijing, China; 5 Frontiers Science Center for Nano-optoelectronics & Collaborative Innovation Center of Quantum Matter, Peking University, Beijing, China; 6 Collaborative Innovation Center of Extreme Optics, Shanxi University, Taiyuan, China; 7 Hefei National Laboratory, Hefei, China

**Keywords:** Photonic crystals, Topological insulators, Nanophotonics and plasmonics, Silicon photonics, Microresonators

## Abstract

Controlling topological phases of light allows the observation of abundant topological phenomena and the development of robust photonic devices. The prospect of more sophisticated control with topological photonic devices for practical implementations requires high-level programmability. Here we demonstrate a fully programmable topological photonic chip with large-scale integration of silicon photonic nanocircuits and microresonators. Photonic artificial atoms and their interactions in our compound system can be individually addressed and controlled, allowing the arbitrary adjustment of structural parameters and geometrical configurations for the observation of dynamic topological phase transitions and diverse photonic topological insulators. Individual programming of artificial atoms on the generic chip enables the comprehensive statistical characterization of topological robustness against relatively weak disorders, and counterintuitive topological Anderson phase transitions induced by strong disorders. This generic topological photonic chip can be rapidly reprogrammed to implement multifunctionalities, providing a flexible and versatile platform for applications across fundamental science and topological technologies.

## Abstract (web summary)

The authors demonstrate a programmable topological photonic chip with large-scale integration of silicon photonic nanocircuits and microresonators that can be rapidly reprogrammed to implement diverse multifunctionalities.

## Main

Topological insulators (TIs) have garnered significant interest because of the abundant physical mechanisms underlying non-trivial bands and potential applications of topological boundary modes^1^. Since the discovery of the quantum Hall effect^2^, the intricate diagrams of topological phases have developed as a sprawling tree with intertwined branches, encompassing dimensionality^3^, symmetry^4^, non-Hermiticity^5^ and defects^6^. One leap recently happened when topology met photonics^7–10^. Photonic systems provide numerous advantages for topological physics and technologies, such as noise-free environment, few constraints on lattice geometry, large diversity of optical materials, high controllability of optical devices and widely adoptable nonlinear optical effects^8–11^. Topological photonics, initially proposed as an extension of topological materials in optical artificial structures, is emerging as an independent field and is revolutionizing optical science and technologies. For examples, integer quantum Hall TIs^12–14^, quantum spin Hall TIs^15,16^, Floquet TIs^17,18^, non-Hermitian TIs^19,20^ and many other interesting topological phenomena have been observed in various photonic systems. Practical topological photonic devices—for example, topological optical delay lines^13^, topological lasers^21,22^ and topological single-photon^23,24^ and entangled-photon sources^25,26^—have been intensively developed and explored.

These observations of topological effects and demonstrations of topological devices are reported on a large variety of optical devices with specifically designed periodic structures or geometries. It is essential to flexibly and precisely control topological phases of light in programmable topological photonic devices at the levels of both fundamental and applied science. First, the dynamics of topological phase transition (TPT) relies on strong reconfiguring of structural parameters of the devices. Topological invariants persist until bands cross so that a marked altering of parameters is required. In typical measurements, TPTs are observed in several different devices, or a joint multivariate effort may even be necessary^27–29^. Though TPTs can be enabled by globally tailoring the devices with an adoption of nonlinear effects^20,30,31^ or mechanical displacement^32^, portrayal of TPTs using more direct and accurate approaches is demanded. Individually programming each artificial atom as well as the atom–atom interactions may represent the ultimate control of the system. This however remains challenging in many natural and artificial topological systems, and also in photonics. Second, most previous observations of topological phenomena rely on static analysis of single or several samples. Comprehensively certificating topological robustness by statistical measurements, and probing interesting statistical topological phenomena such as topological Anderson insulators (TAIs)^33–35^ and amorphous TIs^36,37^, requires the ability to individually programme artificial atoms and their interactions to control disorder. Fabricating a large number of samples with precisely controlled disorder for such statistical analysis is impractical. Third, as topology in matter derives from the collective behaviour of atoms in the lattice, the lattice geometry determines the interrelationships between neighbouring atoms and the overall topological properties. The topology of bands varies in dimensions^3^, and lattices with various geometries also give different symmetries^4^, resulting in TIs in different classes. Previous investigations of TIs in diverse lattices however rely on completely different samples, which necessitates custom design and fabrication of samples.

Programmable photonic integrated circuits^38–40^ across a broad spectrum of advanced optical waveguide materials have been recognized as highly controllable, dynamic and scalable platforms. These are impactful in the realms of both fundamental science and practical applications, including telecommunication and interconnection^41,42^, optical information processing^43,44^, light detection and ranging^45,46^ and quantum information processing and communication^47–49^. Unlike conventional linear-optical circuits that allow merely forward operations of classical^43,44,50^ and quantum^47,48,51^ states of light, a new variety of recirculating photonic networks^52–54^ that allow both forward and backward operations of light has been proposed and demonstrated, marking a departure from previous models. These recirculating photonic circuits intrinsically integrate two types of key photonic component—interferometers and resonators, hence serving as a genetic platform for photonic classical and quantum science technologies. In particular, these systems may even support the emulation of rich topological physics in quantum materials in a fully programmable manner.

In this work, we report a highly programmable topological photonic chip. The chip has generically integrated a lattice of large-scale silicon photonic nanowaveguide circuits and microring resonators, and is fabricated using complementary metal–oxide–semiconductor processes. When we consider each ring as an artificial atom, our photonic chip can be regarded as an artificial lattice that allows arbitrary individual control of atoms as well as the coupling strength and hopping phase between atoms. The generic chip can be rapidly reprogrammed to implement different functionalities: for example, to dynamically transform topological phases of Floquet TIs, observe statistical topological phenomena (statistical analysis of topological robustness and topological Anderson phase transitions) and realize a diverse class of TIs with various lattices (for example, one-dimensional (1D) Su–Schrieffer–Heeger TIs, and two-dimensional Floquet TIs in square and honeycomb lattices). Our work prototypes a flexible, versatile and instantly reprogrammable topological photonic platform.

**Fig. 1:**
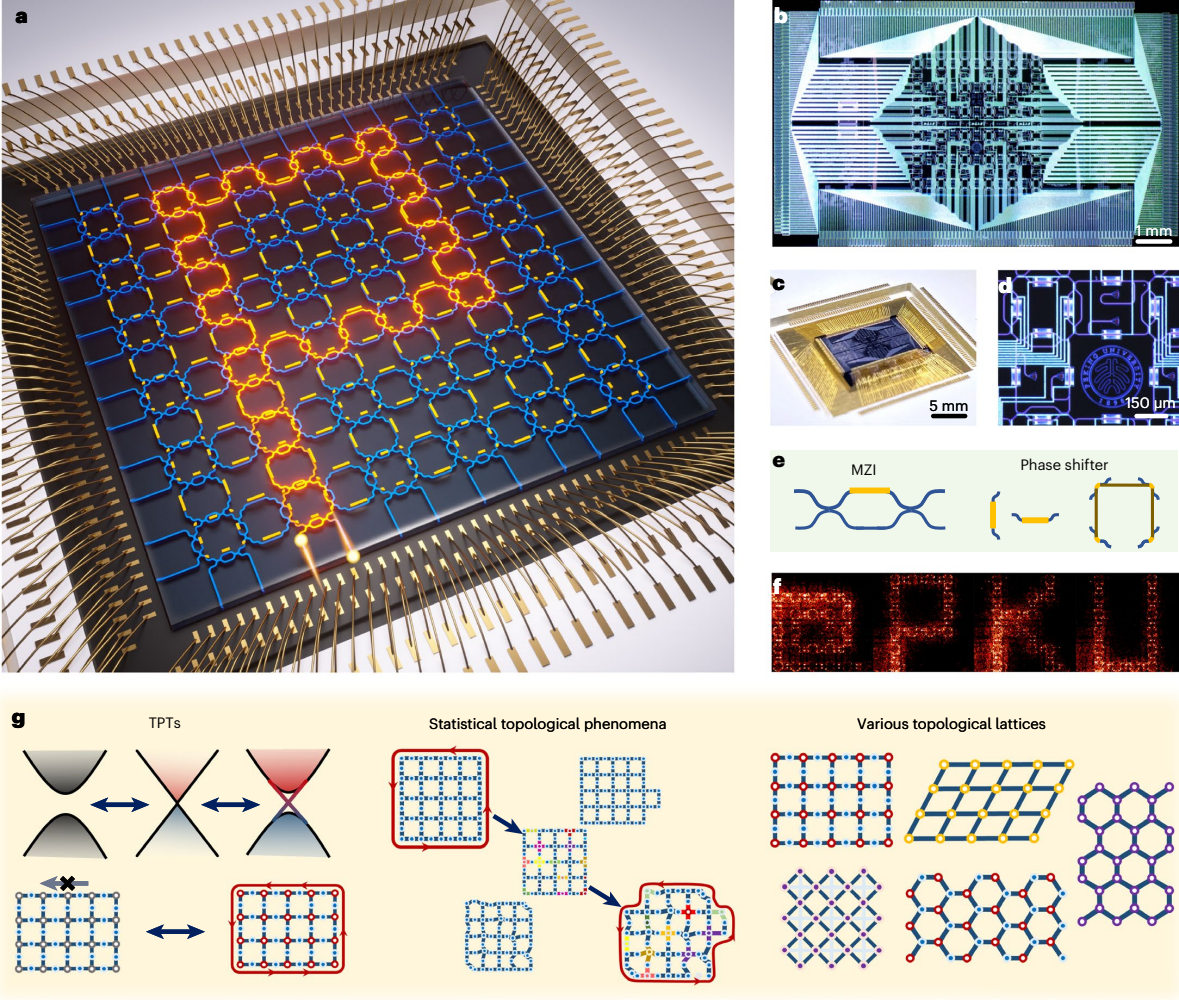
A fully programmable topological photonic chip. **a**, Conceptual diagram. It integrates large-scale photonic nanowaveguide circuits and microring resonators. In total, 96 microrings with high quality factors are regularly positioned in a square lattice of six unit cells by six unit cells. All rings (artificial atoms) can be individually controlled by integrated thermo-optical phase shifters (gold parts), achieving arbitrary resonant phases in all rings, phase differences between the two paths of link rings and coupling strength between neighbouring rings. At the boundaries, 24-in-by-24-out ports are connected to the lattice. **b**, Photograph of a fabricated topological chip. The silicon chip is fabricated using complementary metal–oxide–semiconductor processes and it monolithically integrates 2,712 components in an 11 mm × 7 mm footprint, including 408 low-loss directional couplers, 300 phase shifters with 528 thermal isolators, 48 grating couplers for optical access, 120 tapping ports for light field imaging and 600 electronic access and 708 transmission lines. **c**, Photograph of a packaged chip. The chip is wire bonded on a multilayer printed circuit board (PCB). External electronic drivers with 600 channels are used to individually control 300 phase shifters. **d**, Optical microscopy image of a three-ring unit cell. **e**, Diagrams of reconfigurable optical components, including MZIs and phase shifters. **f**, Imaging of real-space distributions of electromagnetic field. As an example, the chip is flexibly reprogrammed to display @PKU. **g**, The generic chip is reprogrammed to implement multifunctionalities: dynamic TPTs, observation of statistical topological phenomena and benchmarking of TIs in various lattices.

Figure 1 illustrates an overall concept. The photonic topological insulating chip is devised on a two-dimensional lattice of coupled-microring resonators and nanophotonic circuits, as shown in Fig. 1a. Our topological chip is based on recirculating photonic circuits with unique capabilities of operating light states both forwards and backwards^52–54^. One microring emulates one atom, a Mach–Zehnder interferometer (MZI) emulates tunable atom–atom interaction and the photonic chip emulates the artificial atom lattice. In experiment, we use a square lattice of six unit cells by six unit cells, embedding a total of 96 microrings with identical perimeters of 1,234.4 μm, each of which has an intrinsic quality factor of the order of 10^5^. The propagation loss of the silicon nanowaveguides is 2.4 dB cm^−1^. As shown in Fig. 1e, the resonance of each microring can be individually controlled, and the coupling between microrings (both strength and phase) can be arbitrarily controlled by MZIs with an ultrahigh extinction ratio of about 50 dB and ultralow loss of about 0.07 dB. Each phase shifter is thermo-optically driven by 30 mW power for 2π modulation, and accessed by electronic routing circuits. Characteristics of microrings and interferometers such as loss and crosstalk are provided in Supplementary Notes 2.1 and 2.2. The device operates at the wavelength of 1,525 nm. The device properties (for example, loss and power consumption) fall within the average range for silicon photonic technologies at this wavelength. From the technological perspective, the demonstration of the topological chip is the result of fabrication, controlling and packaging of large-scale photonic circuits, and the completeness of measurements. One fabricated and packaged chip is shown in Fig. 1b–d. The high-level controllability and programmability of the generic photonic chip enable sophisticated implementations of dynamic TPTs, statistical topological processes and diverse topological lattices (Fig. 1g). As an initial test of the flexible and fast programmability of the generic chip, Fig. 1f shows imaged field distributions of ‘@PKU’ symbols, and Supplementary Video 1 shows real-time modulations of the letters ‘HELLO’.

**Fig. 2:**
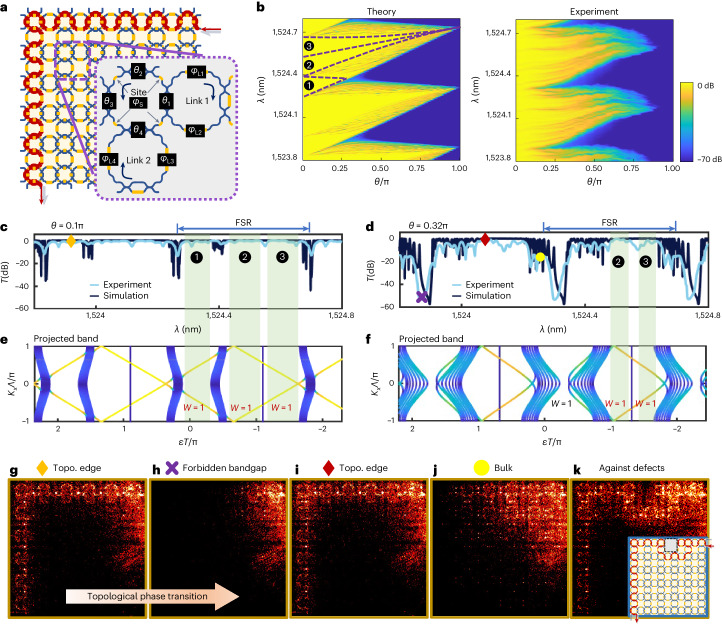
Coupling-strength-controlled TPTs in Floquet TIs. **a**, A three-ring model for Floquet TIs. Using nine parameters in a single unit cell, it fully describes the quasienergy band structure. **b**, TPTs driven by coupling strength. Transmission spectra as functions of wavelength *λ* and the parameter *θ* on coupling strength are shown, where *θ* is negatively correlated with the coupling strength and the amplitude transmittance of an MZI is cos(*θ*/2). The boundaries of non-trivial bandgaps in one FSR are indicated by purple dashed lines. Theoretical results are in good agreement with experimental results. Boundary states at bandgaps ❶ disappear with a continuous variation of *θ* near the critical point at *θ* = 0.272π, while edge modes in bandgaps ❷ and ❸ exist throughout the entire range of *θ* variation. The attenuation of light for large *θ* in experiment is due to resonant enhancement in rings, which increases the effective optical length and thus the loss. **c**–**f**, Measured spectra at *θ* = 0.1π (strong coupling, **c**) and *θ* = 0.32π (weak coupling, **d**) and their respective calculated band structures (**e,f**). The windows of edge modes are visually enhanced. **g**–**j**, Imaged real-space distributions of electromagnetic field under different points marked in spectra in **c**,**d**: TPT from topological edge modes (**g**) to forbidden bandgaps (**h**) at bandgap ❶, edge modes at bandgaps ❷ in weak-coupling regime (**i**) and randomly distributed bulk mode from the non-degenerate bulk bands (**j**). **k**, A boundary cell in Floquet topological insulators (FTIs) is removed by adjusting its coupling to the ‘bar’ state, which forms a lattice defect. High-transmission topological edge modes bypass the hole and present its robustness against atomic vacancies. Note that on the link ring paths we tapped out −35 dB light using diffractive grating couplers for better imaging of light fields, which results in the appearance of regularly distributed bright spots. Noise at the top right arises from light reflection from the input fibre.

We first report arbitrary controls of the band structure of Floquet TIs in the three-particle model, which has been proposed to study non-Hermiticity in Floquet TIs^55^. The famous Floquet theory provides an effective temporal approach for TIs with no need to truly break time-reversal symmetry^18,56^. Demonstrating the full modulation capability requires comprehensive controls of structural parameters, which remain experimentally exclusive. A zoom-in view of a three-ring unit is shown in Fig. 2a and the real structure is shown in Fig. 1d. By reconfiguring four parameters on the coupling strength (*θ*_1−4_) and five parameters on the phase (*φ*_L1−L4_ and *φ*_S_) in a three-ring unit cell, arbitrary topology in the three-band structure can be constructed on the basis of the Floquet band theory. We experimentally characterize two types of Floquet TPT, which are driven by the coupling strength (*θ*) and resonant phase (*φ*_S_), respectively.

For *θ*-driven TPTs, simultaneously tuning all coupling parameters *θ*_1−4_ = *θ* and across the TPT critical point (*θ*= 2 arcsin(√2 − 1) ≈ 0.272π), the bandgaps close and reopen, resulting in disappearance of the topological edge modes (indicated by ❶ in Fig. 2b) and the phase transition at bandgap ❶ from a topological phase to a trivial phase. The topologically invariant winding number (${{{\mathcal{W}}}}$) is used to explicitly portray the topology (that is, ${{{\mathcal{W}}}}=1$ for the non-trivial phase, while ${{{\mathcal{W}}}}=0$ for the trivial phase). Topological invariants are also intuitively reflected in transport properties. Figure 2b shows the theoretical and experimental transmission spectra with a fine tuning of *θ* from 0 to π (that is, transmittance of MZIs from 1 to 0). The flat and high-transmission regimes (outlined by dashed lines) indicate topological edge modes in one free spectral range (FSR). One FSR corresponds to one 2π/*T* period in quasienergy *ϵ*, where *T* is the period of Floquet evolution. Figure 2c,d shows two measured spectra before and after the TPT point, corresponding to the calculated projected bands plotted in Fig. 2e,f. At certain typical points in the spectra, real-space distributions of electromagnetic fields are imaged using an infrared camera (see examples in Fig. 2g–j). Figure 2g,h records light distributions before and after TPTs, while Fig. 2i displays an always existing edge mode at bandgaps ❷. In bulk modes, light dissipates into the bulk (Fig. 2j). Topological immunity against structural defects is tested in Fig. 2k, where one cell is removed by adjusting its coupling to the bar state forming a lattice defect. This indicates an unique ability to withstand and tolerate structure defects. Our topological chip could provide fertile ground for studying the critical conditions for the emergence of defect-induced states^6^.

**Fig. 3:**
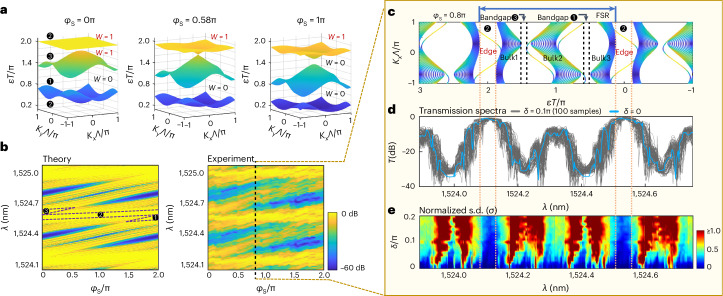
Resonant-phase-controlled TPTs and statistical verification of topological robustness. **a**, Calculated band structures in *φ*_S_-controlled TPT. Starting from the weak coupling regime (*θ* = 0.4π), by increasing *φ*_S_ from 0 to π, site rings and link rings become detuned, reaching a maximum detuning at *φ*_S_ = π. TPT occurs at bandgap ❸ when *φ*_S_ = 0.58π, making it a trivial forbidden bandgap. **b**, Theoretical and experimental transmission spectra as functions of *λ* and *φ*_S_. As a global phase shift is introduced by *φ*_S_, TPT occurs at bandgap ❶. The mapping of bandgaps changes from {❶, ❷, ❸} to {❸, ❶, ❷} after a 2π evolution of phase *φ*_S_. **c**, Calculated projected band structures at *φ*_S_ = 0.8π, plotted as a reference to demonstrate the robustness of topological edge modes. **d**,**e**, Experimental verification of topological robustness with statistical measurements by individually controlling the phase disorders in all rings. The generated random phase obeys the same uniform distribution in the range of *δ*[−0.5, 0.5]. **d**, A set of 100 samples with uniformly distributed random phases is chosen at *δ* = 0.1π in measurement. Measured transmission spectra for the disordered devices are shown as the grey background, and the spectrum for an ideal device without disorder is plotted as a blue line. In the topological edge modes the flat plateaus with high transmission are only slightly influenced, while in all other regimes severe broadening and small dips owing to obstruction from random local modes appear. **e**, The measured s.d. (normalized) of transmission spectra under different strengths of disorder. Evident windows with low fluctuations correspond exactly to the regimes of topological edge modes. *δ* increases from 0 to 0.2π with an interval of 0.01π. S.d. is colour coded and the key is provided at the bottom right.

For *φ*_S_-driven TPTs, in typical Floquet TIs, introducing local phase modulations is challenging, owning to the globally consistent Floquet period in the time domain. On our chip, Floquet TPTs also can be realized by finely altering *φ*_S_ in all the site rings. In Fig. 3a, by turning *φ*_S_ from 0 to π, we continue to reduce the number of non-trivial bandgaps in one FSR from two to one in the *φ*_S_-TPT (when we set *θ* = 0.4π and phase in link rings *φ*_L1−L4_ = 0). Band deformations and changes of topological invariants are shown in the calculated band structures. Bandgaps ❸ become trivial after the critical point *φ*_S_ = 0.58π. As there is a 2π period on resonant phases, it is expected that the spectrum will return to its original state when *φ*_S_ = 2π. Consequently, there must be another TPT to regenerate topological edge modes at the forbidden bandgaps when increasing *φ*_S_ from π to 2π. Figure 3b displays consistent spectra between theory and experiment, showing the disappearance of edge states at bandgaps ❸ within [0, π] evolution and the re-emergence of edge states at bandgaps ❶ within [π, 2π] evolution. Interestingly, the seemingly negligible 2π phase in site rings in fact leads to a reversal of band structure and a global phase shift to lower quasienergy that corresponds to longer wavelength. That being said, the non-trivial bandgaps ❷ and ❸ at *φ*_S_ = 0 correspond to non-trivial bandgaps ❶ and ❷ at *φ*_S_ = 2π, respectively.

Robustness, as the most intriguing property of topological edge modes, allows protection of transport immune to imperfections. As long as the presence of disorder does not interrupt the band structure and the bandgaps remain open, the topological invariants are always constant and light transport along the edge modes is robust. This property has led to many potential applications^13,21–26^. Previously, single or several samples are fabricated, sometimes together with numerical simulation, to verify topological robustness. By harnessing the individual programmability, we experimentally validate the robustness of topological edge modes by statistical measurements. Random perturbations on resonant phases with a uniform distribution of *δ*[−0.5, 0.5], in which the probability density is 1/*δ*, are added to all microrings. We consider a non-trivial device with an initial configuration of *φ*_S_ = 0.8π; see its band structure in Fig. 3c. A set of 100 samples with precisely controlled disorder at *δ* = 0.1π is generated and tested on a single chip. The collections of these statistical measurements (grey lines) are shown in Fig. 3d. The spectrum for an ideal device with no disorder (blue line), in which the topological edge modes in bandgaps ❷ are wide and flat high-transmission plateaus, is plotted for comparison. In the presence of disorder, the high-transmittance plateaus exhibit only small fluctuations in topological edge modes, but large fluctuations in bulk modes. We then estimate the normalized s.d. of transmittance over 100 samples for different levels of disorder (Fig. 3). With these statistical measurements, the observation of low-noise windows for topological edge modes unambiguously confirms the topological robustness against a certain degree of disorder. Moreover, despite the presence of crosstalk when operating a large-scale photonic chip (Supplementary Note 2.3), such inherent topological robustness provides substantial protection. This chip is inherently protected against crosstalk that may take place in real controls and fabrication disorder that may occur in a clean room.

**Fig. 4:**
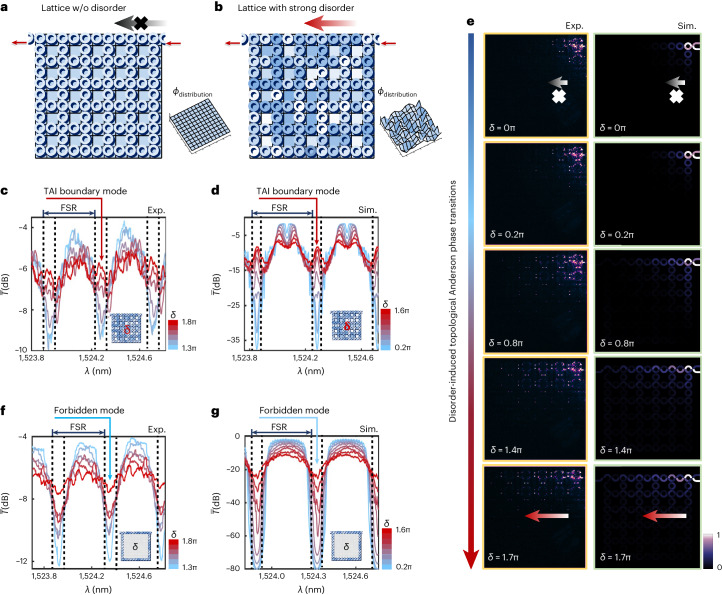
Observation of topological Anderson phase transitions with statistical measurements on individually programming the TI with strong disorder. **a**, Ideal Floquet TI in weak-coupling regime(*θ* = 0.3π) with no disorder. All microrings are matched to resonance. There is a forbidden bandgap within one FSR. **b**, TAI induced by strong disorder. Topological Anderson phase transitions occur at the forbidden bandgaps and connect them with TAI boundary modes. Statistical measurements of the TAI with a large variety of disorder are necessary to observe the topological Anderson phase transitions, which are realized on a single device by individually controlling phase disorder in all rings in our experiment. **c**,**d**, Measured (**c**) and simulated (**d**) transmission spectra of the TAI with different levels of disorder. For each level of disorder, 100 samples are generated on the chip for statistical measurements, and the mean spectrum is plotted. With an increase of disorder, an intriguing peak that represents the TAI boundary mode (indicated by the red arrow) gradually emerges at the low-transmission dip where the forbidden bandgap used to be in the ideal lattice with no disorder. **e**, Imaging the dynamic process of topological Anderson phase transitions. Each image is an accumulated field distribution of 100 samples. The phase transition from a forbidden mode to a TAI boundary mode transported along the upper boundary is observed. Simulation results are shown for comparison, and are in good agreement with experimental results. In contrast to random diffusions of bulk modes, the TAI boundary modes propagate along the boundary and rapidly decay into the bulk. **f**,**g**, Measured (**f**) and simulated (**g**) transmission spectra of a 1D trivial device. The shape of the transmission spectra does not change with increasing disorder, and the low-transmission dip corresponding to the forbidden bandgap remains a dip (indicated by the blue arrow); that is, no phase transition occurred.

Despite the superiority of topological transport, strong disorder may lead to marked deformation of bands and even disrupt the band topology, but this does not mean the properties of the original TI will completely disappear. Interestingly, under specific conditions, the unidirectional transport of the boundary states will still occur in the presence of strong disorder or even amorphous structures^33–37^. Exploring order within areas of disorder is the charm of topology, which particularly requires a highly programmable platform with individual controllability. Recently, the emergence of counterintuitive TAIs from trivial phases has been successfully observed, by inducing sufficiently strong areas of disorder in one sample^33^. Similar to Anderson localization^57^, topological Anderson insulation is also a statistical phenomenon for waves in disordered lattices. Such statistical measurement and verification of TAIs have not been reported in optical systems, to the best of our knowledge. Figure 4b illustrates the random phase distribution in the TAI lattice in the presence of strong disorder. We first consider an ideal lattice in the absence of disorder; *θ* is set as 0.3π, constructing one trivial bandgap within one FSR. We are interested here in the bandgaps that used to be forbidden, that is, the dips of blue spectra in Fig. 4c,d. Experiment and simulation results of averaged transmission spectra over 100 samples with different levels of disorder in the resonant phase are reported in Fig. 4c,d, respectively. A peak gradually emerges at the windows of forbidden bandgaps (indicated by the red arrow) as the strength of disorder reaches a sufficiently large value, indicating the occurrence of topological Anderson phase transitions. The emergence of the TAI phase can be portrayed by real-space topological invariants^18,58,59^. Analogous to the winding number ${{{{\mathcal{W}}}}}_{\epsilon }$ in momentum space, the real-space ${{{{\mathcal{W}}}}}_{\mathrm{real}}$ related to non-trivial bandgaps approaches unity, while it fluctuates around zero for trivial bandgaps. According to the averaged ${{{{\mathcal{W}}}}}_{\mathrm{real}}$ in Supplementary Fig. 23, a non-zero plateau obviously arises from the ordinary zero dip in forbidden bandgaps. Moreover, Fig. 4e shows the imaged real-space field distributions with an increase of disorder, each of which is an overlaid distribution of all 100 samples for better characterization of the dynamics of phase transitions. The TAI boundary modes break free from the localization near the input, and unidirectionally move along boundaries with an exponential decay into the bulk lattice. In contrast, the same measurements were conducted in trivial coupled resonators of optical waveguides (CROW) (Fig. 4f,g inset). The shape of the spectra remains unchanged and no TAI boundary modes are observed, as shown in Fig. 4f,g.

**Fig. 5:**
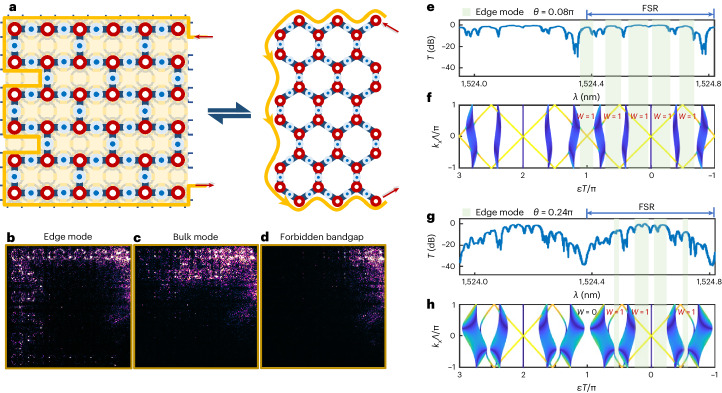
Floquet TIs in the honeycomb lattice on reconfiguring the lattice geometry. **a**, A reconfigured square lattice that is equivalent to the honeycomb lattice. **b**–**d**, Measured real-space distributions of light field in different modes: topological edge mode (**b**), bulk mode (**c**) and forbidden bandgap (**d**). **e**,**f**, Measured transmission spectrum (**e**) and calculated projected band (**f**) when the device works at *θ* = 0.08π. There are five bulk bands in one FSR, distinct from the three-particle model in a square lattice. The winding numbers ${{{\mathcal{W}}}}$ in all bulk bandgaps are unity, implying the existence of non-trivial boundary modes. **g**,**h**, Measured transmission spectrum (**g**) and calculated projected band (**h**) when the device works at *θ* = 0.24π. When decreasing the coupling strength to the critical point of *θ* = 0.19π, TPTs occur at the bandgap across *ϵ* = π/*T*, turning it into a trivial forbidden bandgap (${{{\mathcal{W}}}}$ = 0), while other bandgaps remain non-trivial.

We further benchmark photonic TIs in various lattice structures. Experimental results for the well known Su–Schrieffer–Heeger 1D TIs are shown in Supplementary Fig. 20. The redundant dimension for the 1D models in a two-dimensional lattice allows observations of the non-Hermitian skin effect in Supplementary Fig. 21, and other unordinary experiments such as non-reciprocity and next-nearest-neighbour coupling are implementable. Moreover, it is also possible to achieve other two-dimensional lattice geometries beyond the inherent square lattice by reprogramming microrings. Figure 5 illustrates an example of equivalent Floquet TIs in the honeycomb lattice. A perfect correspondence between the measured transmission spectra and simulated projected band structures in the strong-coupling regime (*θ* = 0.08π) and weak-coupling regime (*θ* = 0.24π) is shown in Fig. 5e–h. When *θ* is larger than 0.19π, TPTs occur at the bandgaps across *ϵ* = π/*T* and the flat high-transmission plateau turns into a blocked dip. Distinct real-space field distributions for different modes are shown in Fig. 5b–d, including topological edge modes conducting along the honeycomb boundaries, dissipatively distributed bulk modes and inhibitively forbidden bandgaps. By distinguishing the winding number, we observe phase transitions in a five-bulk-band structure. Such multiple non-trivial topological phases in standard honeycomb lattices have been achieved in another recent work^60^, using chain-driven laser-written waveguides. This effectively validates the correctness and reliability of our programmable topological chip. In addition, the results show that, by reconfiguring the device, a squared mesh-based TI can be equivalently translated into a TI based on the hexagonal mesh^53,54^. Such topological equivalence is also indicated in a scheme shown in Supplementary Fig. 25.

This work has demonstrated a flexibly and rapidly programmable topological photonic chip. Multifunctionalities are benchmarked by reprogramming the generic chip, including dynamic TPTs, realizations of diverse topological lattices and implementations of statistical measurement of topological processes. Our generic chip could be directly used to discover topological phases of light and understand exotic phenomena. The chip possesses unique backward operations in a large-scale lattice of optical resonators and it may provide an alternative solution for classical^38–40^ and quantum^47–49^ information processing and computing tasks. It could provide flexible hardware to model the lattice of topological materials and predict their physical properties. Such reprogrammability could even allows dynamic simulation of real-world materials, where disorder, inhomogeneity and anisotropy are commonly present. To emulate these complex topological materials and matter, a larger-scale lattice is required. Further scaling of the topological chip is achievable by delicate design of the recirculating photonic circuits and electrical routing circuits. Advanced silicon-based integrated photonic technologies may provide the ultimate solutions for very-large-scale integrated programmable TIs, using heterogeneous integration of different optical materials on silicon^61^ and photonics–electronics packaging or co-integration^62^. Programmable topological photonic chips may provide one generic platform for fundamental science and topological technologies.

## Online content

Any methods, additional references, Nature Portfolio reporting summaries, source data, extended data, supplementary information, acknowledgements, peer review information; details of author contributions and competing interests; and statements of data and code availability are available at 10.1038/s41563-024-01904-1.

### Supplementary information

Supplementary Information Supplementary Figs. 1–25 and Discussion.

Supplementary Video Real-time modulation of the on-chip optical patterns to form a ‘HELLO’.

## Data Availability

The data that support the findings of this study are available from the corresponding authors upon reasonable request.
