# Supplementary material for: A programmable topological photonic chip
Source: Nat Mater. 2024 May 22;23(7):928–36. doi: 10.1038/s41563-024-01904-1 (PMC11230904; doi:10.1038/s41563-024-01904-1)
Supplement: Supplementary file 1 — Supplementary Figs. 1–25 and Discussion. [file 41563_2024_1904_MOESM1_ESM.pdf]

nature materials

Supplementary information

https://doi.org/10.1038/s41563-024-01904-1

A programmable topological photonic chip

In the format provided by the authors and unedited

Table of contents

Supplementary Note 1: Fabrication of silicon-photonic topological chip

Supplementary Note 2: Characterisations of the programmable topological photonic chip
2.1: Device details and experimental setup
2.2: Calibrations of MZIs and microrings
2.3: Characterisations of local and global thermal crosstalk

Supplementary Note 3: Three-particle Floquet topological insulators

Supplementary Note 4: Observations of CROW chain, SSH model and non-Hermitian skin effect in 1D lattices

Supplementary Note 5: Topological Anderson insulators in a Floquet lattice

Supplementary Note 6: Scheme of a programmable topological photonic chip based on a hexagonal lattice

Supplementary Note 7: The scalability of programmable topological photonic chip

Supplementary Note 1: Fabrication of silicon-photonic topological chip

The programmable topological photonic chips were fabricated on a silicon-on-insulator wafer using standard complementary metal-oxide-semiconductor (CMOS) processes and 248 nm deep ultraviolet (DUV) lithography processes. The devices were in-house fabricated in "Integrated Circuit Advanced Process Centre" in the Institute of Microelectronics of Chinese Academy of Sciences. The wafer has a buried oxide layer of 3 μm thickness and a top silicon layer of 220 nm thickness. First, a thin oxide layer was formed on the wafer by thermal oxidation. The wafer was coated with a positive photoresist. Full and shallow etching structures were patterned by DUV lithography, and such patterns were then transferred to the Si layer by double inductively coupled plasma (ICP) etching processes. The full-etched waveguides have a fully etched depth of 220 nm and they form the topological coupled ring lattice, beamsplitters and MZIs, whereas the shallow-etched waveguides have an etched depth of 70 nm and they form the grating coupler. A special annealing process was performed to smooth the sidewall of the device. Subsequently, a layer of 1-μm-thick cladding oxide was deposited by plasma-enhanced chemical vapour deposition. A 10-nm-thick Ti glue layer, 20-nm-thick TiN barrier layer, 800-nm-thick AlCu layer and 20-nm-thick TiN anti-reflective layer were subsequently deposited by physical vapour deposition, and they were patterned by DUV lithography and etching processes to form the electrode. A 50-nm-thick TiN layer for the thermo-optical phase shifter was deposited and patterned. Finally, a thin layer of oxide was deposited to protect the device, and the bonding pad window was opened for wire bonding.

Supplementary Note 2: Characterisations of the programmable topological photonic chip

Supplementary Note 2.1: Device details and experimental setup

The topological photonic chip bases on a squared mesh of 6-by-6 high-Q microrings (in total 96 identical microrings). The highly tunable microring resonators and nanophotonic circuits in the lattice are shown in Fig.1d,1e. Operating the topological chip with fully programmability and high fidelity relies on a collaborative operation of the squared recirculating optical lattice in topological configurations. Figure 1e in main text or Figure S2a shows a packaged topological photonic chip, that is wire-bonded on a PCB. Figure S1 shows the schematic of experimental setup. Silicon nanowaveguides were designed and fabricated with a cross-section of 450 nm $\times$ 220 nm, and the propagation loss of silicon waveguides was measured to be 2.4 dB/cm. The topological ring arrays are woven together by site rings and link rings. A site ring consists of four low-loss MZIs and a corner-distributed phase shifter. And a link ring is formed by two MZIs and two length-compensated phase shifters, which control not only the resonant phase of link rings but also a nonreciprocal jumping phase between neighboring site rings. Both the site rings and link rings have an identical perimeter of 1234.4 μm to satisfy the requirement of Floquet theory. The MZIs were designed with two low-loss directional couplers (DCs), in between a phase shifter is adopted to tune the phases. The insertion loss of a single MZI is 0.07dB, see measurement methods in Fig.S5. Figures S4a, b illustrate the wavelength response of transmittance and reflectance of an individual MZI at crosser state and bar state, respectively. With the variation of wavelength, the splitting ratio of the DCs deviates from 50:50. As shown in Figs.S4c-f, the gradual deviation from the perfect crosser state leads to a topological phase transition. Phase shifters were designed with 2μm width, 100μm length, and 20nm thickness, and formed by resistive TiN materials having a resistance of 600 Ohms. The electronic routing circuits were made of 800nm-thick AlCu with a minimum width of 5μm, to avoid resistive heating there. The use of thermo-optical phase shifters limits the upper bound of modulation speed to the kilohertz (kHz) range. To avoid electronic crosstalk, every phase shifter has its own signal and ground accesses (in total, 600 electronic routing circuits and electronic pads). To reduce thermal crosstalk and to enhance the thermo-optical tuning efficiency, we developed the deep trench etching process to etch the silicon dioxide cladding on both sides of each phase shifter, which can efficiently isolate the thermal field with the surrounding dissipation channels (see Supplementary Note 2.3). The topological chip was optically accessed by two arrays of 24/24 optical input/output (I/O) ports, coupled to single-mode fiber arrays through on-chip grating couplers. Moreover, light was tapped out for efficient imaging of the light field in the topological chip, by using DCs with a weak coupling efficiency (-35dB) positioned along the paths of link rings in clockwise quasi-spin and by diffracting light out of plane using grating couplers. A continuous wave (CW) laser light at the wavelength of around 1525nm was used to charaterise the chip and implement topological photonic experiments. For example, Fig.S3b shows the measured power transmission of one MZI, and an ultra-high extinction ratio over 62dB is characterised in Fig.S3c. The extinction ratio of MZIs in three rows and three columns was tested, respectively (Fig.S3a), to demonstrate the ultra-high modulation capabilities over the whole device. Detailed information of the calibrations of phase shifters is provided in Supplementary Note 2.2. We note that the device properties, such as waveguide losses and power consumption for 2π-modulation (shown in Fig.S8), are average for silicon photonic technologies at this wavelength range. It is the execution of the photonic circuits, controllers, packaging, and the completeness of the measurements that stand out from a technological point of view.

Imaging system: Figure S1 shows an optical microscopic imaging system. We used an infrared CCD to capture the scattered light so as to directly image the distributions of light fields in the topological device. The collected light for an infrared image (those shown in Fig.2 and Fig.5) come from three main sources: scattered light from the waveguides, spots from the tapped-out grating couples for detections, and light reflections from the input fiber. Light propagating along waveguides is scattered in all directions due to the random roughness of waveguide surface, while only vertically scattered light can be captured by the CCD. In our device, the electric routing layer on the top of the optical waveguide layer partially blocks the scattered light. Therefore, the scattered light from waveguides that captured by the CCD is relatively weak. Since the partially scattered light from waveguides is rather weak, we designed tapped-out GCs in all the connection in the link rings, in order to more precisely identify the field distributions on the chip. Less than 0.1% light has been tapped out by evanescent coupling and vertically emitted by GCs. Because of its directionality, the light spots can be clearly observed, which can unambiguously indicate the light field distributions. These method give a visualized and accurate feedback about the distributions of light fields, especially for the regions of dense electrical routing and weak optical pump. Note that our topological chip supports optical excitation in both quasi-spins, but only one direction (CCW in the site and CW in the link) is selected for a better visual presentation. Moreover, part of light from the input fibers is not yet coupled into the photonic chip, but reflected on the top surface of the chip. Because the direction of reflected light is nearly vertical to the chip surface, this part of light is also captured by the CCD and introduces strong noises to the infrared imaging. In our experiment, the GCs was optimized for the 1550nm wavelength at an incident angle of 10 degree. However, the optimal wavelength for directional couplers was

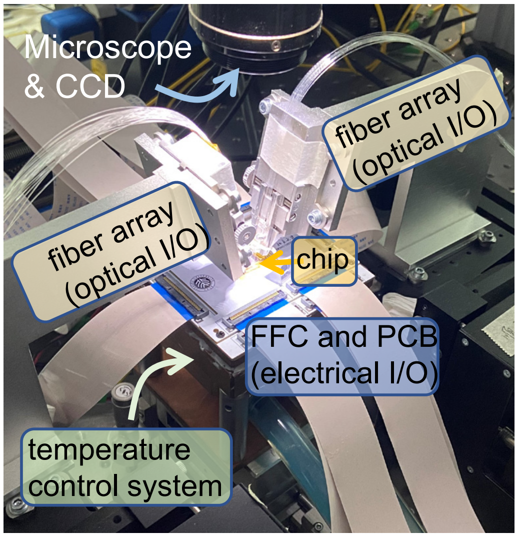

Fig. S1 Experimental setup. The topological photonic chip is wire-bonded on a printed circuit board (PCB, see photo of a packaged chip in Fig.S2a) and positioned on a temperature-controlled stage, on which temperature is controlled and stabilised with an assistance of a thermoelectric cooler (TEC) system and a water cooling system. Two arrays of 24/24 optical input/output (I/O) ports are coupled to single-mode fiber arrays through on-chip grating couplers. A number of 300 integrated thermal-optical phase shifters are routed out via electronic routing circuits to the boundaries of the chip, and wired bounded to the PCB. All these channels are connected to electronic drivers/controls through flexible flat cables (FFCs). An optical microscopic imaging system (including a visible light CCD and an infrared CCD camera) on the top of the chip is used to nearly real-time image the real-space distribution of light field of the topological chip.

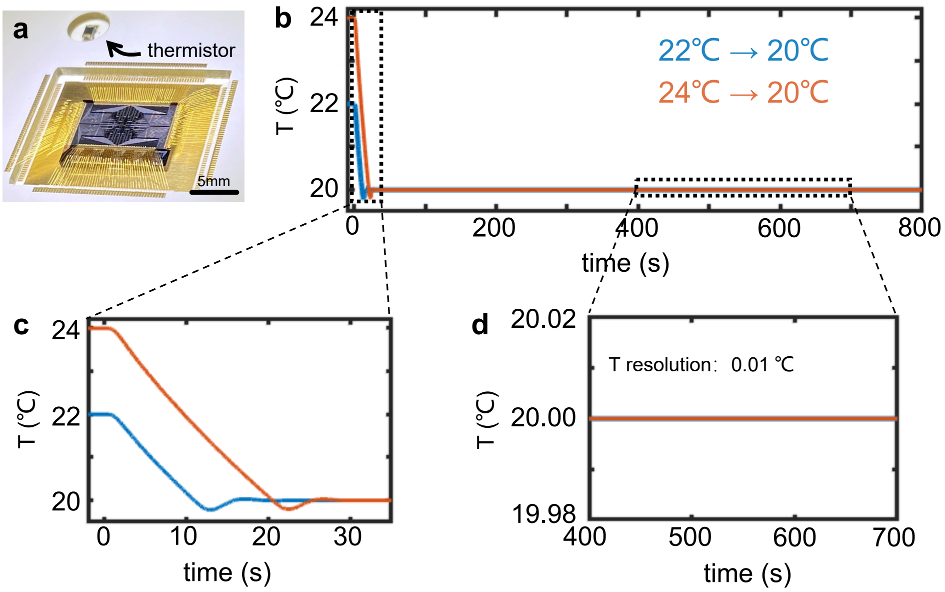

Fig. S2 Temperature control of the topological photonic chip. **a**, Photograph of a packaged chip. A thermistor is positioned on the PCB near by the chip. It can feedback the temperature of the chip in real-time. A number of 600 pads positioned around the boundaries of chip (electronic routing circuits are used to connect the 300 phase shifters to the pad array) is wire-bonded on the PCB. **b**, Measured temperature variations, when the target temperature varies from 24°C or 22°C to 20°C, respectively. **c**, and **d**, Zoomed-in view of temperature variations. The temperature rapidly is stabilized within less than 30s, reaching a cooling rate of approximately 2°C/10s. After temperature stabilization, the fluctuation is less than 0.01°C, limited by the system's detection accuracy.

near 1525nm, at which the coupling loss for GCs was about 6.8dB. To reduce those noises, it is necessary to improve the coupling efficiency between fibers and GCs. Alternatively, replacing the input GCs by edge couplers that allowing side coupling of light (parallel to the top surface of chip) with low loss and much wider bandwidth, could provide a better solution of suppressing noises.

Temperature controlling system: Optical devices especially microrings are phase-sensitive to environment drifts such as temperature variations. The total power consumption of the programmable topological photonic chip can fluctuate with different parameter configurations, especially during the initial loading bias, where the power can change from 0mW to approximately 4W in a short time. While changes in the ambient temperature are relatively slow, overcoming phase shifts caused by environmental temperature fluctuations is crucial for the stable operation of the chip. Therefore, we equipped the chip with a stable PID temperature control system. As shown in Fig.S1, the topological chip wire bounded onto an aluminum PCB is positioned on a thin copper block on top of a TEC, that is connected to a water cooling system. By controlling the voltage and current of the TEC, it is convenient to either heat or cool the chip. The temperature of the topological

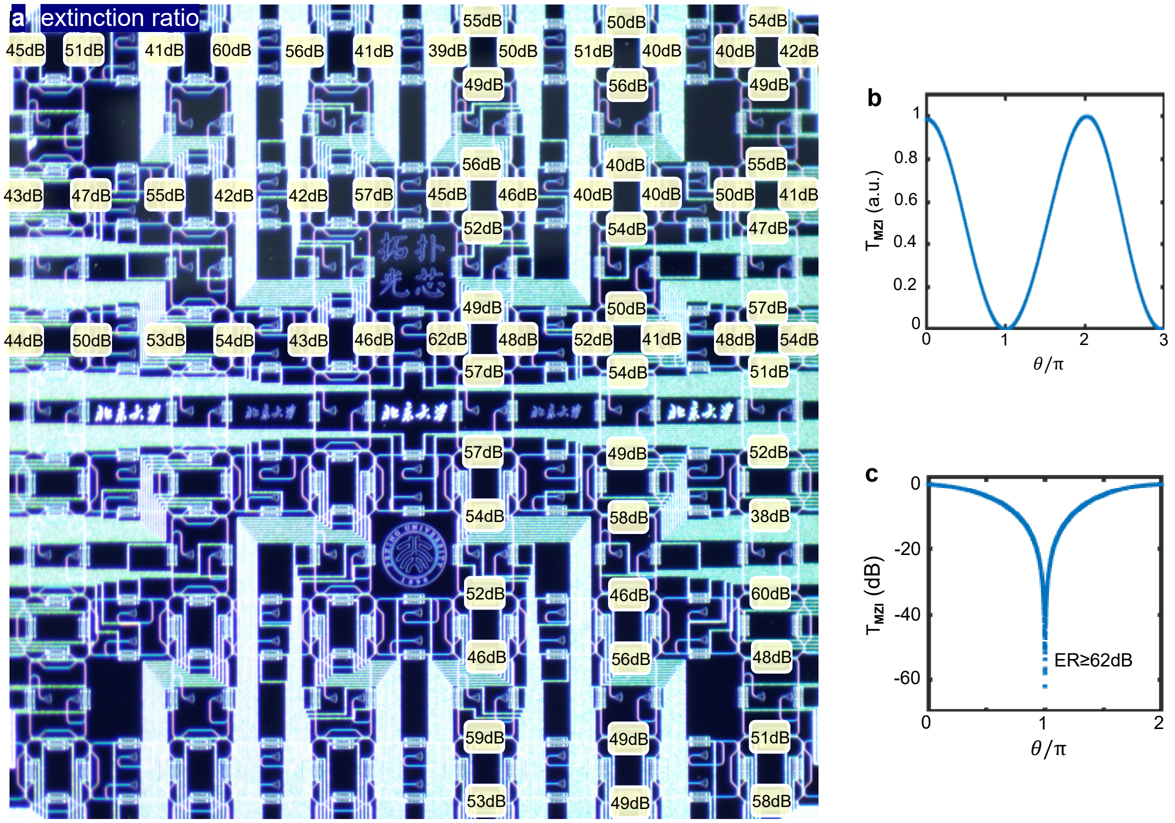

Fig. S3 Characterisations of extinction ratio of MZIs in the lattice. **a**, Extinction ratios of MZIs sampled in three rows and three columns at around 1525nm. **b**, Measured cosine-shape power transmission of MZIs modulated by θ. **c**, Power transmission of MZIs in logarithmic scale for extinction ratio measurment.

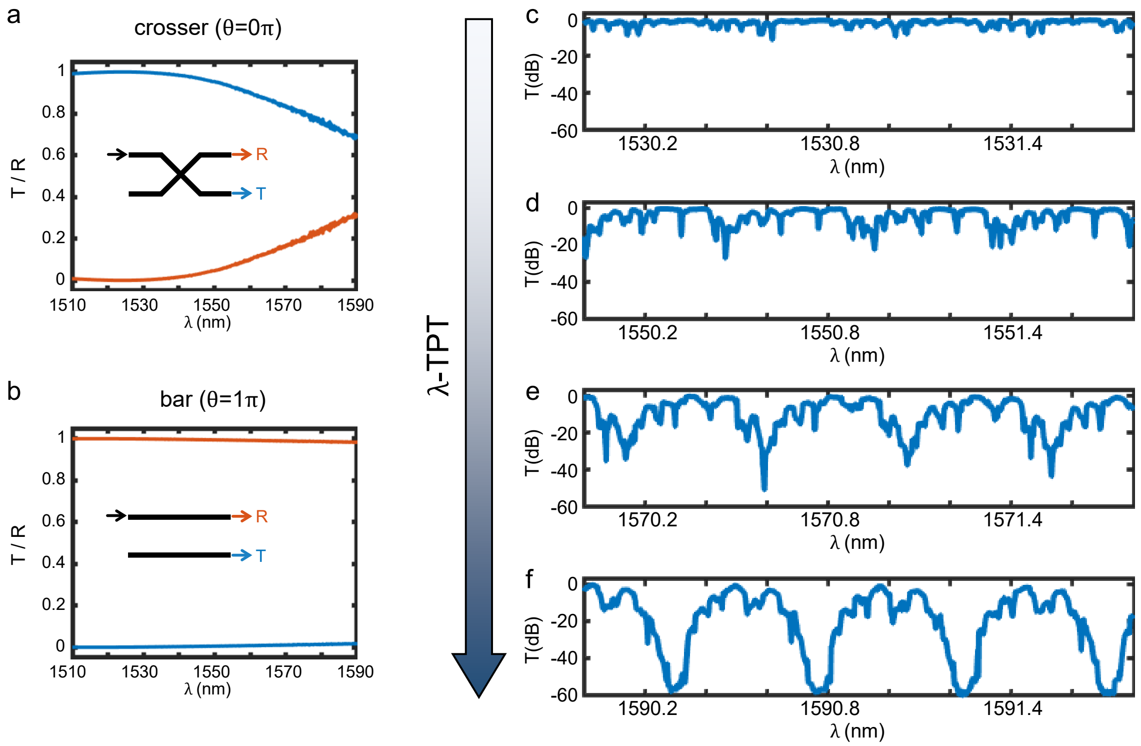

Fig. S4 Experimental wavelength response of components and the device. Wavelength response of the transmittance and reflectance of the MZI in the (**a**) cross state and (**b**) bar state. The narrowing of the MZI modulation range is mainly due to the gradual deviation from the ideal cross state, while the bar state is almost unaffected. **c-f**, λ-induced TPTs from a AFTI to CI. All θ in the lattice except at the boundary are set to zero.

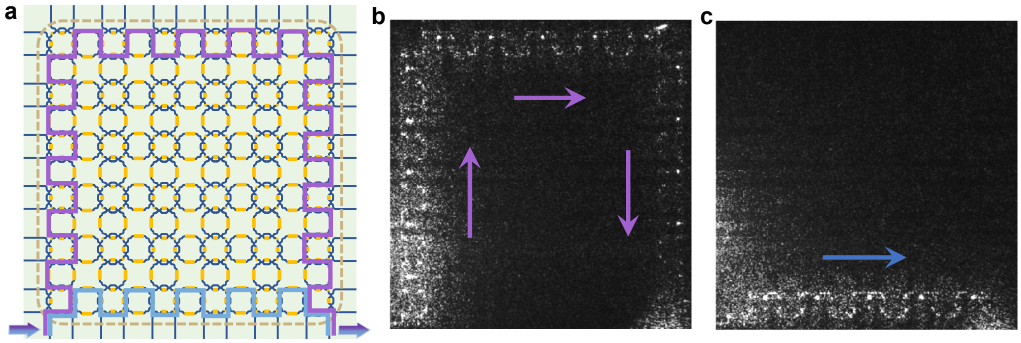

Fig. S5 Characterisations of the insertion loss of MZIs. **a**, A scheme for characterising the insertion loss of MZIs. Two optical circuits with the same optical input and output ports but different lengths (passing a different number of MZIs) are programmed by setting the relative MZIs to bar states ($\theta = \pi$) or crosser states ($\theta = 0$). As the perimeter of a single ring is $1234.4\mu m$, the length difference between the two paths is $12.7\mu m$. **b**, and **c**, Real-space distributions of light fields for the long route and short route, respectively. The power difference between the two imaged paths is measured to be 5.8dB. This results in an averaged propagation loss of 4.6 dB/cm, including propagation losses in waveguides (2.4 dB/cm) and insertion losses of MZIs (0.07dB/MZI).

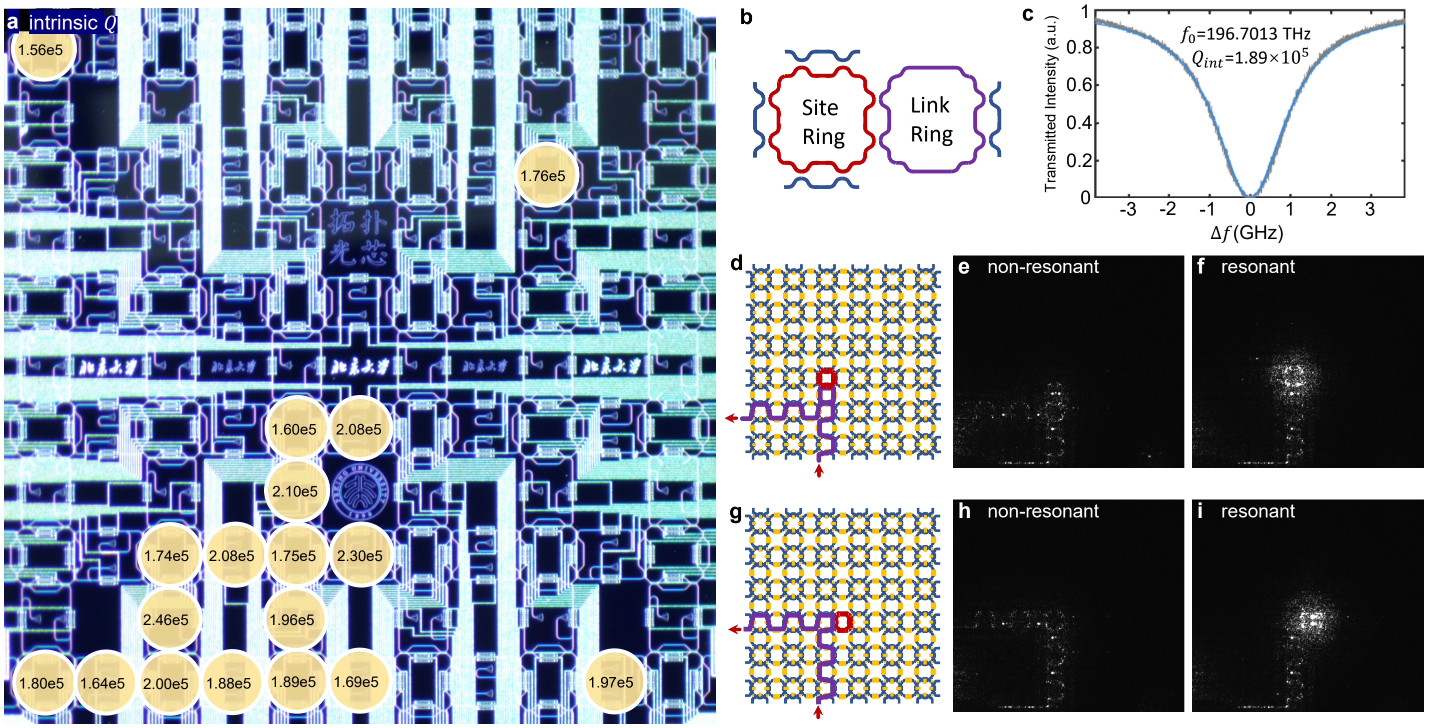

Fig. S6 Characterisations of quality factor of microrings in lattice. **a**, intrinsic Q factors of sampled microrings. According to the symmetry, Q_{int} of rings in an eighth of area and some randomly selected microrings were measured, reaching a level of 2×10^5. **b**, Schematic of the geometric bendings of waveguides in site rings and link rings. The uninterrupted closed waveguides provides the assurance of high Q factor. **c**, Transmission spectrum of a ring resonator under critical coupling at 1525nm. **d** and **g**, optical routing for Q factor measurment of a site ring (link ring). The red ring is the ring for measurment. And the purple bus waveguide is achieved by properly setting "crosser" and "bar" states of MZIs. Real-space distributions of electromagnetic field at (**e** and **h**) non-resonant states and (**f** and **i**) resonant states are measured. Light are strongly confined in resonant modes and the optical field in the cavity is obviously enhanced.

chip is monitored in real-time using a thermistor near the chip, as shown in Fig.S2a. A PID control system feed-backwardly adjusts the voltage of the TEC based on real-time temperature and the target temperature of the chip. This system stabilizes the chip temperature at the set point with a precision of 0.01 degrees Celsius. In Figs.S2b,c, while the chip was operating in typical configurations, we lowered the chip's temperature from 24 or 22 degrees Celsius to 20 degrees Celsius, respectively. The chip's temperature was rapidly stabilized within less than 30s, reaching a cooling rate of approximately 2 degrees Celsius/10s. Subsequently, the chip's temperature stably remained at 20 degrees Celsius, with fluctuations below the system's detection accuracy of 0.01 degrees Celsius. When there is a significant change in the total power consumption on the chip, the chip's temperature experiences slight fluctuations but quickly stabilizes again. In our experiment, we accordingly adjust the target temperature of the chip based on changes in thermal power consumption to achieve phase compensation. Further details are provided in Supplementary Note 2.3.

Furthermore, we measured the intrinsic quality factor (Q_{int}) of a single ring. Typical routings for Q measurment are presented in Fig.S6d and g. The "S"-shape bus waveguide (purple lines) and the test ring (red rings) are equivalent to a microring coupling to a single waveguide, by properly setting the splitting states of relative MZIs to "crosser" or "bar" states. Benefitting from the low-loss design of MZIs, both the site

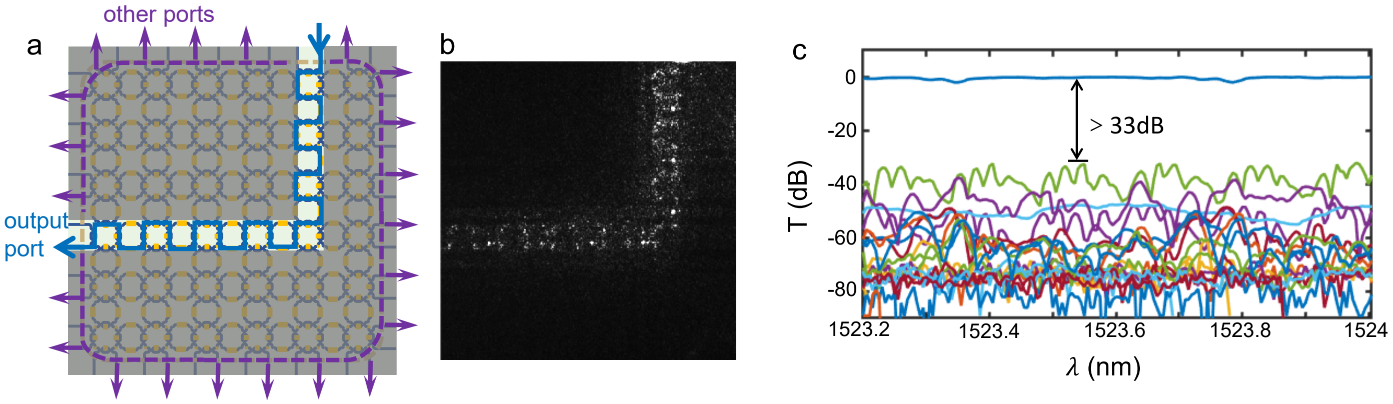

Fig. S7 Characterisations of the optical crosstalk. a, A scheme for characterising crosstalk, when light is propagating along the constructed optical channel by reconfiguring the lattice. Light intensity at the output port (blue arrow) as well as all other ports (purple arrows) are collected. **b**, Infrared image of light field, using the reconfiguration in (a). **c**, Measured (normalised) optical transmission spectra in all ports indicated in (a). A low optical crosstalk of around -33dB is observed between the target output port and other ports.

rings and link rings are complete rings without any interruptions in the middle as Fig.S6b shown. Therefore, all the rings exhibit extremely high quality factors for silicon chips. The transmission spectra under critical coupling and the fitting curve are shown in Fig.S6c. Strong resonant field enhancement effect can be observed in infrared imaging (Fig.S6e,f and h,i). Fig.S6a presents representative statistics of Q_{int} around 2×10^5, which is already a very high level for integrated silicon photonics.

For an initial characterization of the chip's collaborative performance, we constructed a simple optical path as shown in Fig.S7a and tested the optical crosstalk at all output ports. Figure S7b,c show the imaged light field and measured crosstalk. The optical crosstalk is less than -33 dB. Despite the good optical performance achieved, the extinction ratio is somewhat reduced compared to a single MZI. The main reason is the thermal crosstalk among heaters when operating collaboratively. A detailed analysis of thermal crosstalk is provided in Supplementary Note 2.3. The collaborative implementation of the optical and electronic layers enables excellent control and modulation of optical intensity, phase and frequency. These outstanding performances satisfy the requirements of various topological models and ensure that the integrated topological photonic chip serves as a highly controllable platform to probe topological physics.

Supplementary Note 2.2: Calibrations of MZIs and microrings

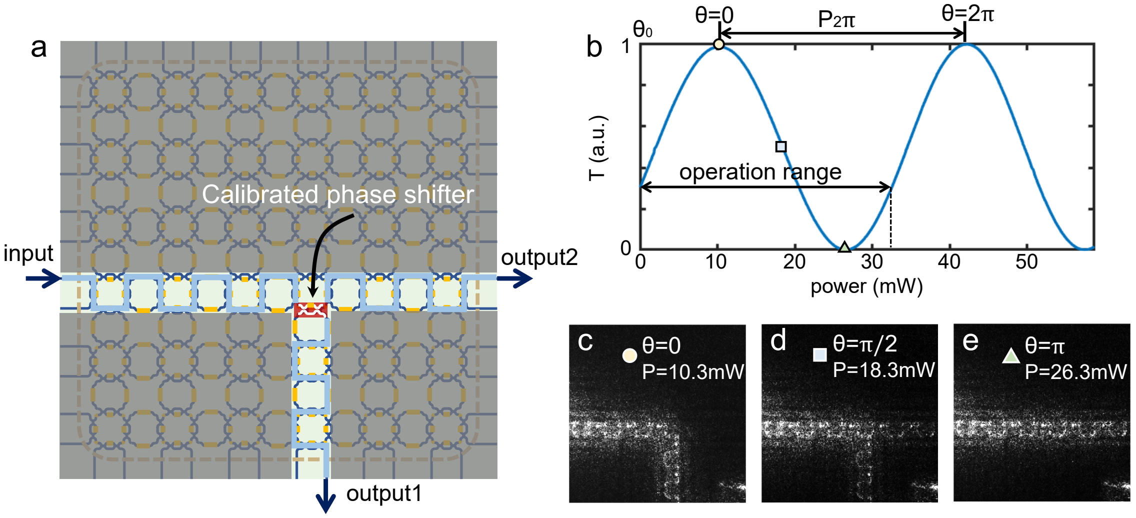

Fig. S8 Calibrations of optical phases in MZIs. a, A scheme for calibrating a target MZI. The MZIs along the blue paths are set to be working at the crosser states or bar states. In this case, the cosine-shaped linear response to the power added on the phase shifter can be observed at the output ports 1 and 2. **b**, Measured (normalized) intensity transmission at the output port1, which is in good agreement of theoretic outcome of $\cos^2(\theta/2)$. The maximal and minimal intensity corresponds to $\theta = 0$ and π, respectively, and it also tells the power consumption for 2π-modulation ($P_{2\pi}$). The initial phase at P=0mW is defined as θ_0. As the parameter θ has a 2π period, the operation range of electronic power in experiments is limited in $[0, P_{2\pi}]$. **c-e**, Measured infrared image of light fields at $\theta = 0, \pi/2$ and π, respectively.

To ensure a given programmed configuration is actually transferred into the topological chip, all phase shifters (PSs) underwent detailed calibrations to guarantee that specific θ and φ were loaded. Here we show the calibration of the PSs in MZIs and the PSs controlling the resonant phases in microrings, respectively. The statistical results of all PSs are provided.

Calibrations of the MZIs: As shown in Fig.S8a, when calibrating the target phase shifter in the MZI (indicated by red), all MZIs along the blue paths are switched to the bar or crosser state. At this point, these blue optical paths only act as straight waveguides. When scanning the

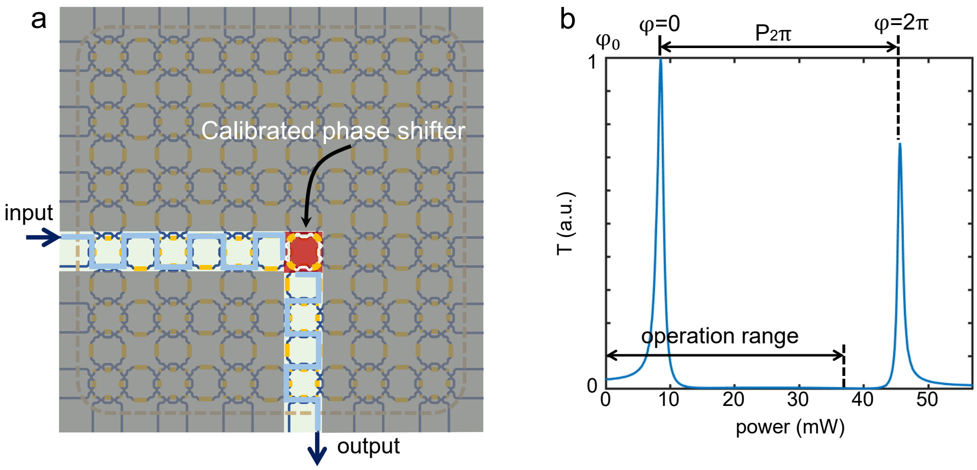

Fig. S9 Calibrations of optical phases in microrings. a, A scheme for calibrating optical phase in the microring. The 4 MZIs in this site rings are switched to the bar state ($\theta = \pi$). The MZIs on blue paths are set to the crosser state or bar state. The wavelength of input light is fixed at 1523.20nm as the reference. **b**, Measured (normalized) intensity transmission at the output port as an increase of power added on the target phase shifter. The two peaks correspond to $\varphi = 0$ and 2π, and $P_{2\pi}$ represents the power difference between the two peaks. The initial phase at P=0mW is defined as $\varphi 0$. As the parameter φ has a 2π period, the operation range of electronic power in experiments is limited in $[0, P_{2\pi}]$.

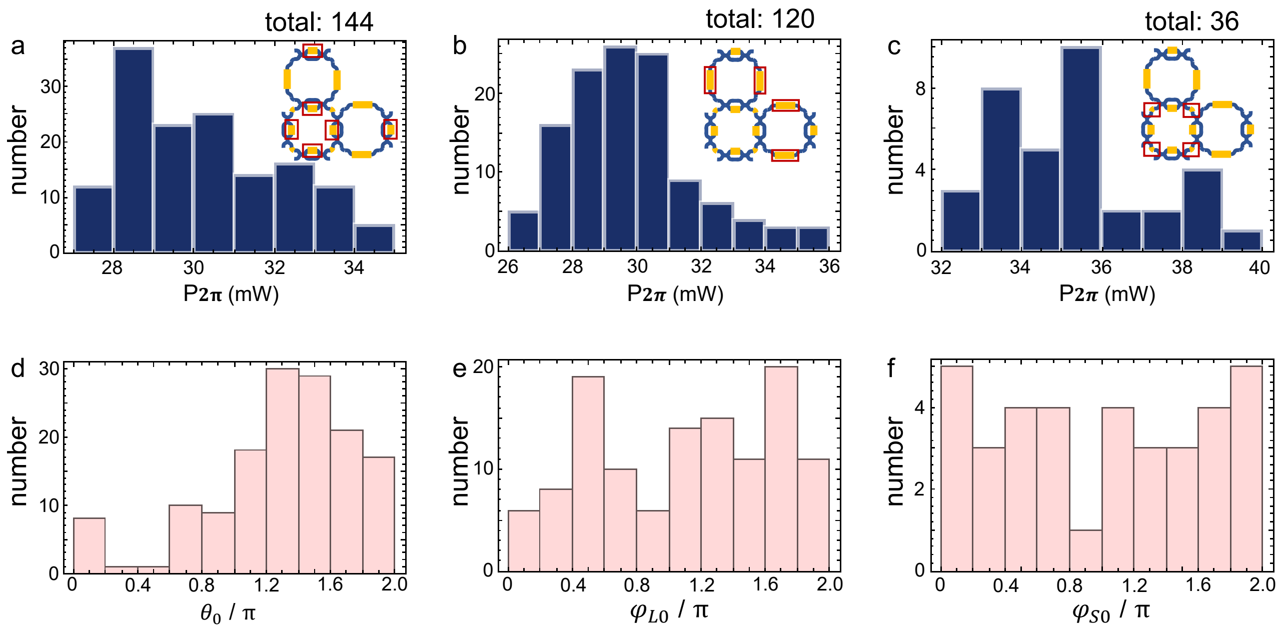

Fig. S10 Histograms of measured 2π-modulation power and the initial phase for all phase shifters. a-c, Histograms of 2π-modulation power for the three types of phase shifters (see insets) in the programmable topological photonic chip. The typical value of $P_{2\pi}$ for phase shifters is about 30mW. **d-f**, Histograms of initial phase at P=0mW for the three types of phase shifters (see insets in (a-c) respectively) in the programmable topological photonic chip. The initial phases of phase shifters are randomly distributed after fabrications.

power added on the target phase shifter, the output intensities of ports 1 and 2 return as complementary cosine functions. Figure S8b shows a measured (normalized) intensity transmission at the output port1, which is in good agreement of theoretic outcome of $\cos^2(\theta/2)$. The maximal and minimal intensity corresponds to $\theta = 0$ or π, respectively, and it also tells the power consumption for 2π-modulation ($P_{2\pi}$). The measured real-space distributions of light fields at $\theta = 0, \pi/2$ and π are shown in Figs.S8c-e, respectively. Moreover, it is important to note, during the initial calibration, all MZI phase parameters are unknown. Calibration should start from the MZIs located at the corners, then proceed to the MZIs along the boundaries of the lattice, and gradually move inward the lattice centre until all MZIs are calibrated once. Subsequent calibration processes require multiple iterations to eliminate potential errors introduced by unwanted optical loops.

Calibrations of the microrings: The calibrations of microrings embedding in the topological lattice are dependent on the calibrations of MZIs first. After several iterations of calibrating all MZIs as Fig.S8, the calibrations of the PSs in intra-ring can commence. As shown in Fig.S9a, all the four MZIs in the site rings are setting to bar states ($\theta = \pi$), and the additional total phase induced by the MZIs in the site ring is 4π, which can be ignored. Note that throughout the calibration process of microrings, the wavelength of the incident light must be fixed, which serves as the phase reference point. Figure S9b shows a measured (normalized) intensity transmission at the output port by scanning the power added on the target phase shifter inside the microring (indicated by red). At the two resonant peaks, the power bias for $\varphi = 0$ and

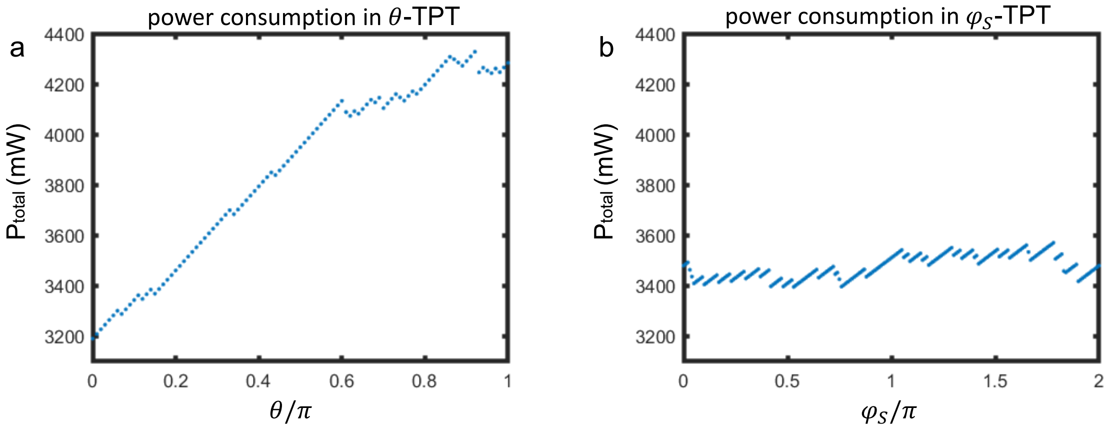

Fig. S11 Power consumption when the chip operates θ-TPT and φ_S-TPT. Measured total power consumption of the chip, when it is reconfigured (all phase shifters are switched on) to implement two types of topological phase transitions, (**a**) coupling strength (θ-TPT) and (**b**) resonant phases in site rings (φ_S-TPT).

$P_{2\pi}$ can be obtained. The calibration process for the phase shifters within the link rings is similar. The calibrations of the phase shifters that control resonant phases of microrings also take multiple iterations. During the calibration process, we set other irrelevant phase shifters to a 0-phase configuration while switching the temperature compensation to avoid thermal crosstalk (see our discussions on crosstalk).

Power consumption: Figure S10 summarizes the $P_{2\pi}$ and the initial phase (θ_0, $\varphi_{S0,L0}$) for all PSs. We have optimized the design of phase shifters and electronic routing. The geometric parameters of the resistive TiN phase shifters were optimized with a resistance of 600 Ohms, and the electronic transmission lines were optimized to avoid resistive heating there. Deep-etched thermal isolators were used to enhance the thermal localisation and reduce thermal crosstalk (see Supplementary Note 2.1). Thus, heat is highly confined near the phase shifters. In Fig.S10, it presents histograms of power consumption ($P_{2\pi}$) for all PSs. The $P_{2\pi}$ for one PS is only 30mW, which indicates a high thermo-optical tuning efficiency. Although the $P_{2\pi}$ for a single phase shifter is already small, the global thermal crosstalk when simultaneously operating all 300 heaters is a lot and cannot be ignored. Fortunately, since the initial phases (after fabrication) for phase shifters are randomly distributed (see Figs.S10 d-f), the total power consumption remains roughly the same when the configuration of the chip is altered. Figure S11 shows the total power consumption (P_{total}) for two different types of TPTs (θ-TPT and φ_S-TPT) reported in the main text. The P_{total} for typical settings of the topological chip is between 3W and 4.5W. We will discuss the temperature stability and how to overcome the impact of global thermal crosstalk in the Supplementary Note 2.3.

Supplementary Note 2.3: Characterisations of local and global thermal crosstalk

The management of crosstalk represents a challenging task when operating a large-scale programmable photonic circuit. In this section, we provide detailed characterizations of the thermal crosstalk. We classify the thermal crosstalk into two categories: local thermal crosstalk and global thermal crosstalk. The former refers to the influence of thermal crosstalk between neighboring or next-neighboring phase shifters, while the latter accounts for the impact of total power added on all phase shifters.

Local thermal crosstalk: Benefitting from the high efficiency of phase shifters and the use of thermal isolation (see Supplementary Note 2.1), the local thermal crosstalk between neighboring phase shifters is limited. Figure S12 shows the measured local thermal crosstalk on one target MZI from its nine neighboring heaters (the main source of local crosstalk). The interference fringes are measured by tuning the phase shifter in the target MZI from 0 to 2π, while the power of the crosstalk sources increases from 0mW to 40mW (larger than $P_{2\pi}$ =30mW). The patterns of all interference fringes in Fig.S12b-j remain almost unchanged with high visibilities, in the presence of local thermal crosstalk from all different neighboring heaters in different directions. Then, we characterised the local thermal crosstalk (resonance drifting) in microrings. Similarly, we measured the drifting of the resonance in the target microring, when the power added on the neighboring microrings increases. An example is shown in Fig.S13. When the power of the crosstalk source increases from 0 to 30mW, the resonant phase only drifts by 0.035π. Moreover, neighboring microrings with different distances were tested, as shown in Fig.S13a. It confirms that the impact of local thermal crosstalk decreases as the distance increase, and even for the strongest case of crosstalk sources, the phase drifting of resonance is smaller than 0.06π within the 0-30mW power range. And the impact of local thermal crosstalk gradually decreases with increasing distance.

Global thermal crosstalk: Although the $P_{2\pi}$ for a single phase shifter is already small, the global thermal crosstalk when simultaneously operating all 300 heaters is a lot and cannot be ignored. Figure S11 shows the total power consumption remains roughly the same when the configuration of the chip is altered. Then, we quantified the impact of global thermal crosstalk by measuring the phase drifting in a microring as the P_{total} varied, when the topological chip is positioned on top of a temperature-stablised stage. As shown in Fig.S14a, the phase drifting is linearly related to the total power consumption, and the phase drifting is less than 1.5π for a full range of P_{total} for typical settings of the topological chip. On the other hand, the phase drifting in microrings can be tuned by changing the temperature of the whole chip. We established a stable and fast temperature control system for the topological chip. Figure S14b shows that the measured phase drifting is also linearly related to the temperature of the entire chip. We obtained a phase modulation over 3.5π by increasing the chip's temperature from 16 degrees Celsius to 28 degrees Celsius. Therefore, the phase drifting induced by global thermal crosstalk from all other on-chip heaters can be elimated by T-compensation on the entire chip. Based on the measured phase offset and its relationship with P_{total} and T, we have now obtained a relationship for phase compensation in terms of chip temperature and total power consumption:

$$T(^\circ C) = -(P_{total}(mW) - 4000) \times 0.0028 + 21 \tag{S1}$$

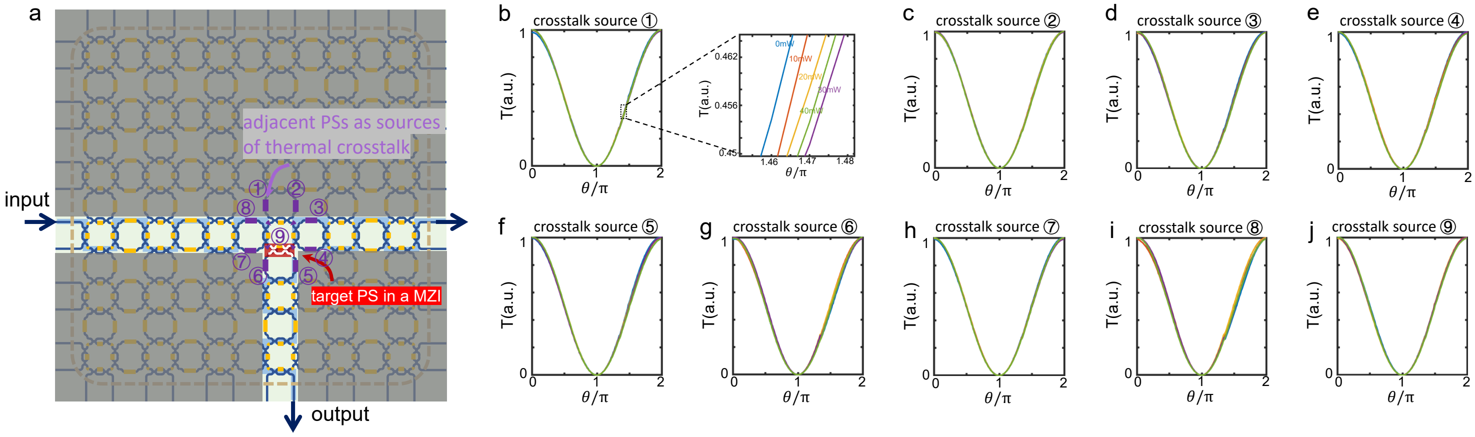

Fig. S12 Characterisations of local thermal crosstalk on MZIs. **a**, A scheme for characterising local thermal crosstalk on a target MZI (red area) from the surrounding thermal sources (indicated by numbers of 1–9). The MZIs on blue paths are set to the crosser states or bar states. The cosine-shaped linear response to the power of target heater can be observed at the output port. We set the power of the local crosstalk sources (indicated by numbers of 1–9) to 0mW, 10mW, 20mW, 30mW, and 40mW, and then collect the interference fringes, respectively. **b-j**, Measured interference fringes of the target MZI, under the perturbation from the same crosstalk source (indicated by numbers of 1–9) but with different powers added on the crosstalk sources. The interference fringes of the target MZI remain unchanged under the influence of nine different local thermal crosstalk sources in different directions.

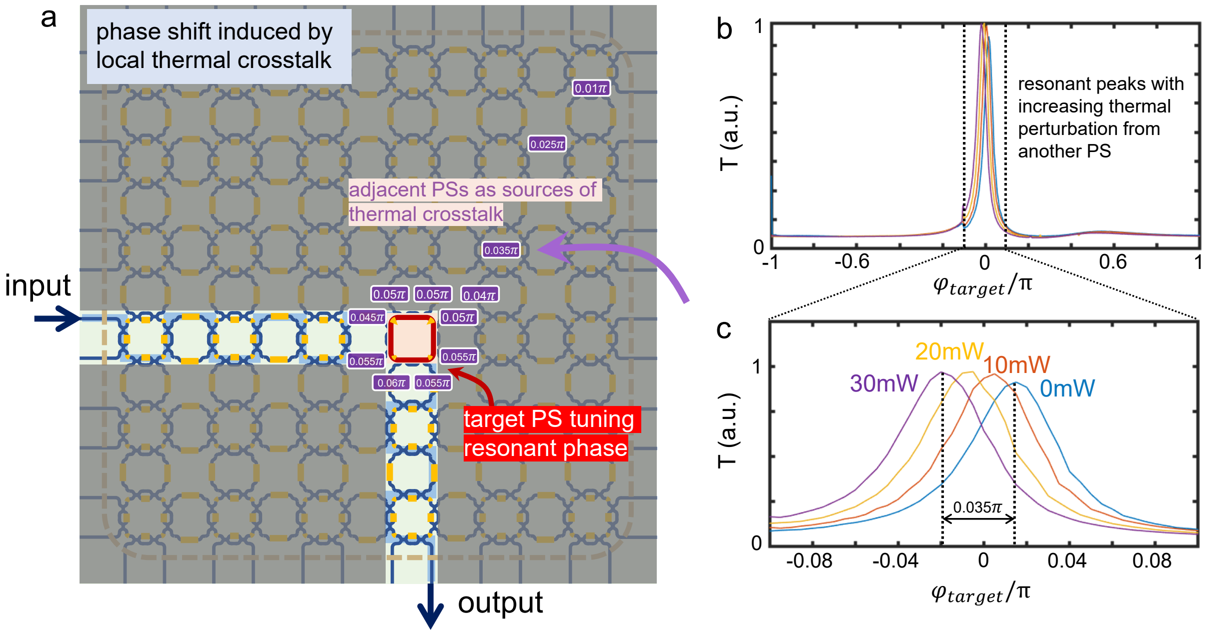

Fig. S13 Characterisations of local thermal crosstalk on microrings. **a**, A scheme for characterising local thermal crosstalk on the target ring (red colored). from the surrounding thermal sources (indicated by purple areas). The MZIs on blue paths are setting to the crosser states or bar states. The resonant phase of the target microring can be tuned by scanning the phase shifter inside the microring, allowing the record of transmission at the fixed wavelength. We set the power of the local crosstalk sources to 0mW, 10mW, 20mW, and 30mW, and then record the phase drifting of the resonant peaks. **b**, and **c**, Measured transmission spectra by tuning φ_{target} in the target ring, when different powers are added on the crosstalk sources. The resonant phase drifting due to local crosstalk is indicated by the arrow (here 0.035π). Measured phase driftings by different local crosstalk sources are indicated in the corresponding purple areas in (a). The phase drifting induced by local thermal crosstalk is smaller than 0.06π, and it decreases rapidly with increasing distance.

A specific set of (P_{total}, T) is presented in Figs.S14c,d. With these parameters, the global thermal crosstalk can be well compensated.

We then recharacterized the influence of global thermal crosstalk, with T-compensation, by measuring the phase shift of microrings and interference patterns of MZIs. The global thermal crosstalk is introduced by increasing the power of all the irrelevant heaters. In Figs. S15a,b, the resonant phase of the target link ring only drifts from 0.026π to -0.014π while the P_{total} increases from 3.35W to 4.47W. We tested the phase shift for a row of rings and their shifts are indicated in Fig.S15c. The drift is well controlled within 0.05π indicating that the global thermal crosstalk has been effectively eliminated. Moreover, the impact of global thermal crosstalk on MZIs is recharacterized. The global thermal crosstalk adds almost identical additional phases to both arms of the MZI, thus not altering the interference patterns of MZIs. We experimentally verified it in Fig.S16. We measured the interference fringes for 6 MZIs locating from the center to the edge of the chip, with increasing P_{total} mentioned in Figs.S14c,d. The solid line represents the mean, and the pink shaded region indicates one standard deviation ($\pm 1\sigma$). In sum, we confirm that by temperature control, the global thermal crosstalk has been well compensated.

Importantly, the topology of the entire photonic lattice is robust against the presence of disorders of resonant phases, as shown in Fig.3 in

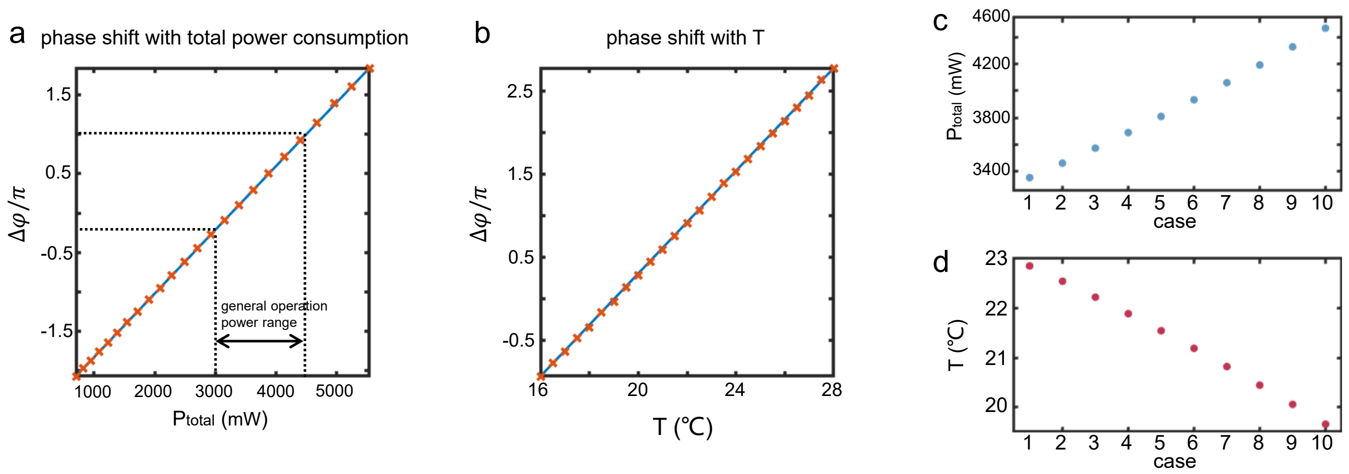

Fig. S14 Characterisations of resonant phases in microrings with different temperature and total power consumption added on the topological chip. a, Measured phase shift in a microring with an increase of total power consumption (P_{total}). The relation of $\Delta\varphi$–P_{total} fits linearly. In this test, P_{total} was chosen within [1W, 5.5W], while in most of typical experiments, the chip was operated within [3W, 4.5W]. **b**, Measured phase shift in a microring with an increase of temperature added on the entire chip. The relation of $\Delta\varphi$–T fits linearly. The global thermal crosstalk induced by P_{total} can be compensated by changing the temperature of the entire chip. **c**, and **d**, A set of T-compensation parameters [P_{total}, T]. It can be estimated by the P_{total}-T equation shown in Eq. S1.

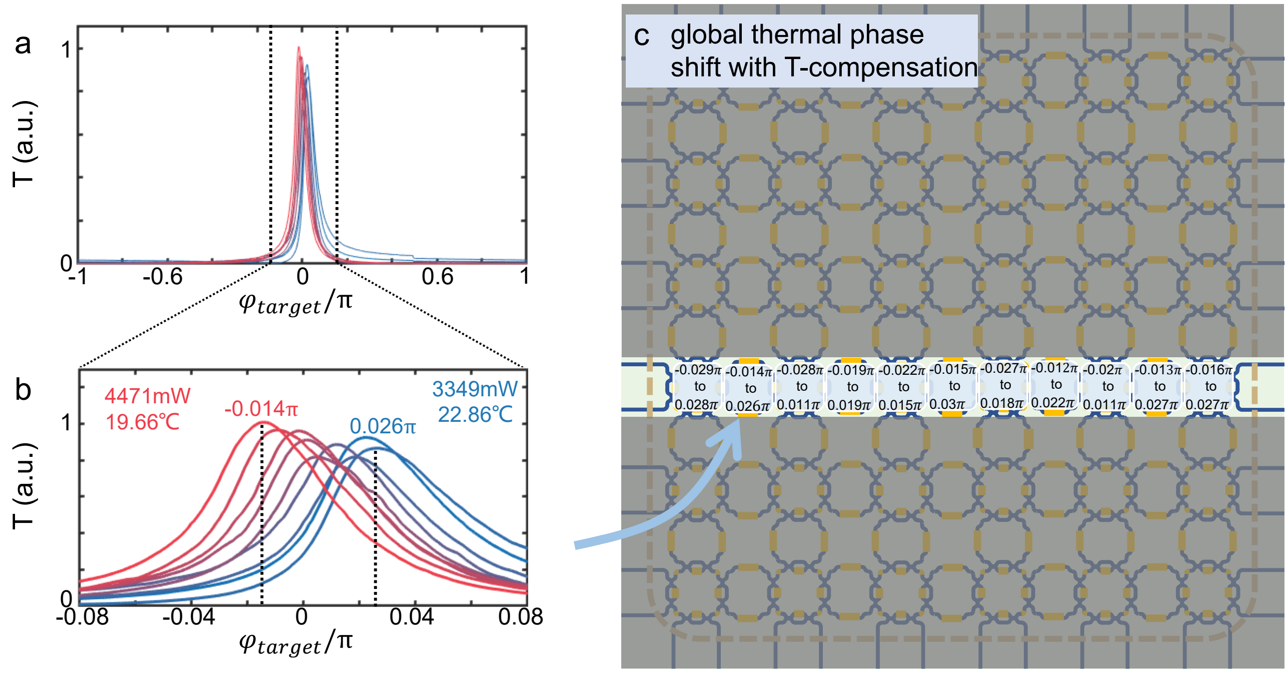

Fig. S15 Characterisations of global thermal crosstalk on microrings with T-compensation. a, and **b**, Measured transmission spectra by tuning φ_{target} in a target ring (indicated by the arrow in (c)), when different strengths of global thermal crosstalk are added on the entire chip. For each measurement, T-compensation is on. That means, the temperature of chip is controlled according to Eq.S1 so as to compensate the global phase shift. After T-compensation, the resonant phase drifting is controlled within $[-0.014\pi, 0.026\pi]$. **c**, Measured resonant phase drifting of a row of microrings. Microrings in a row are measured to quantify the global phase shift at different positions.

main text. This topological robustness can protect our device immune to fabrication disorders as well as crosstalk, those shown here either local or global thermal crosstalk.

Supplementary Note 3: Three-particle Floquet topological insulators

The microring-based Floquet model has become one of the optimal choices for programmable integrated topological insulators, with its flexible and rich band structures, simple and feasible implementation compatible with CMOS process, and clear and accurate theoretical description. The three-particle Floquet topological insulators (FTIs) serve as a primary framework selected in our photonic chips to demonstrate multiple topological phase transitions and statistical topological phenomena. A detailed description about its theory is necessary to understand the model thoroughly. This computational method and characterisation approach are equally applicable to other Floquet models, including all the 1D models in Supplementary Note 4 and honeycomb lattice in the main text.

Ensuring accurate temporal periodicity is crucial for designing FTIs. For our microring-based FTI, it means optical path compensation

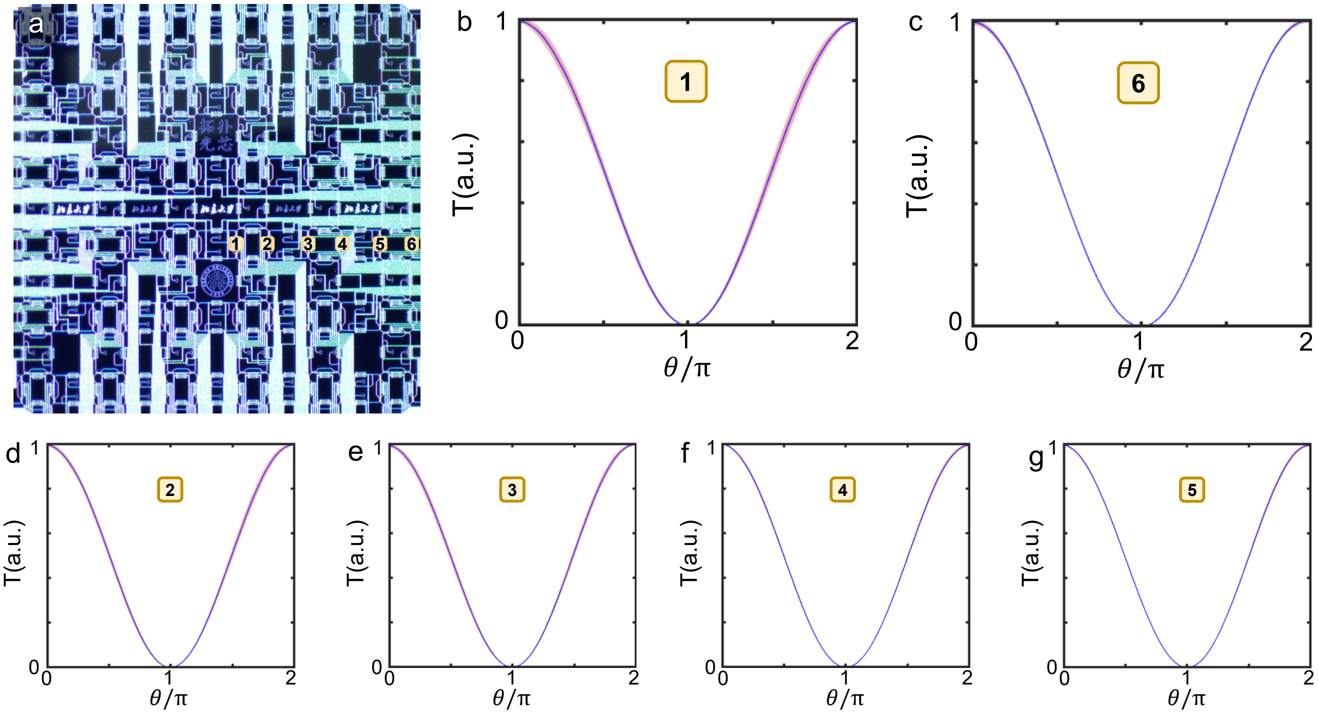

Fig. S16 Characterisations of global thermal crosstalk on MZIs with T-compensation. a, We characterised an array of 6 MZIs in different locations from the center to the edge of the chip (indicated by numbers of 1–6), and quantified the impact of global thermal crosstalk with T-compensation. The interference fringes are plotted in (**b**-**g**), respectively. For each MZI, interference fringes were measured under 10 different cases of global thermal crosstalk with power consumption from 3.5W to 4.5W. The solid line represents the mean, and the pink shaded region indicates one standard deviation ($\pm 1\sigma$).

for the same circumference in site rings and link rings. Then the propagation and interference in the ring array can be perfectly governed under the framework of Floquet theory. Figure. S17b illustrates the Floquet time evolution in a bulk cell in periodic boundary condition. Following the definations of parameters in the main text (Fig.2a), the repeated evolution can be devided into four steps. The evolution of photon states in different steps follows a piecewise Hamiltonian $H_k(t)$. When $nT \le t < (n + T/4)$ in Step 1, for example, only ring a and ring b are coupled and there is only a propagation phase accumulated in ring c, where t is the evolution time, T is the period and n is an integer. The Floquet transfer matrix is calculated as time evolution under the Hamiltonian in a period T,

$$U_F = \mathcal{T}\exp[-i\int_0^T H_k(t)\,dt], \tag{S2}$$

where $\mathcal{T}$ is the time-ordering operator. In order to map to the practical device, the transfer matrix of a MZI is written as,

$$U_{MZI}(\theta) = e^{i(\theta/2+\pi/2)} \begin{bmatrix} \sin(\theta/2) & \cos(\theta/2) \\ \cos(\theta/2) & -\sin(\theta/2) \end{bmatrix} \tag{S3}$$

Next, the Floquet transfer matrix in a Bloch bulk cell can be calculated,

$$\begin{aligned} &U_F(kx, ky, \theta_{1-4}, \varphi_S, \varphi_{L1-L4}) = U_4 \cdot U_3 \cdot U_2 \cdot U_1 \\ &= \begin{bmatrix} e^{i\varphi_{L2}} & 0 & 0 \\ 0 & e^{i(\theta_4/2+\pi/2+\varphi_S/4)}\sin(\theta_4/2) & e^{i(\theta_4/2+\pi/2+\varphi_S/4-k_y\Lambda)}\cos(\theta_4/2) \\ 0 & e^{i(\theta_4/2+\pi/2+k_y\Lambda)}\cos(\theta_4/2) & -e^{i(\theta_4/2+\pi/2)}\sin(\theta_4/2) \end{bmatrix} \\ &\cdot \begin{bmatrix} -e^{i(\theta_3/2+\pi/2)}\sin(\theta_3/2) & e^{i(\theta_3/2+\pi/2+k_x\Lambda)}\cos(\theta_3/2) & 0 \\ e^{i(\theta_3/2+\pi/2+\varphi_S/4-k_x\Lambda)}\cos(\theta_3/2) & e^{i(\theta_3/2+\pi/2+\varphi_S/4)}\sin(\theta_3/2) & 0 \\ 0 & 0 & e^{i\varphi_{L4}} \end{bmatrix} \\ &\cdot \begin{bmatrix} e^{i\varphi_{L1}} & 0 & 0 \\ 0 & e^{i(\theta_2/2+\pi/2+\varphi_S/4)}\sin(\theta_2/2) & e^{i(\theta_2/2+\pi/2+\varphi_S/4)}\cos(\theta_2/2) \\ 0 & e^{i(\theta_2/2+\pi/2)}\cos(\theta_2/2) & -e^{i(\theta_2/2+\pi/2)}\sin(\theta_2/2) \end{bmatrix} \\ &\cdot \begin{bmatrix} -e^{i(\theta_1/2+\pi/2)}\sin(\theta_1/2) & e^{i(\theta_1/2+\pi/2)}\cos(\theta_1/2) & 0 \\ e^{i(\theta_1/2+\pi/2+\varphi_S/4)}\cos(\theta_1/2) & e^{i(\theta_1/2+\pi/2+\varphi_S/4)}\sin(\theta_1/2) & 0 \\ 0 & 0 & e^{i\varphi_{L3}} \end{bmatrix}, \end{aligned} \tag{S4}$$

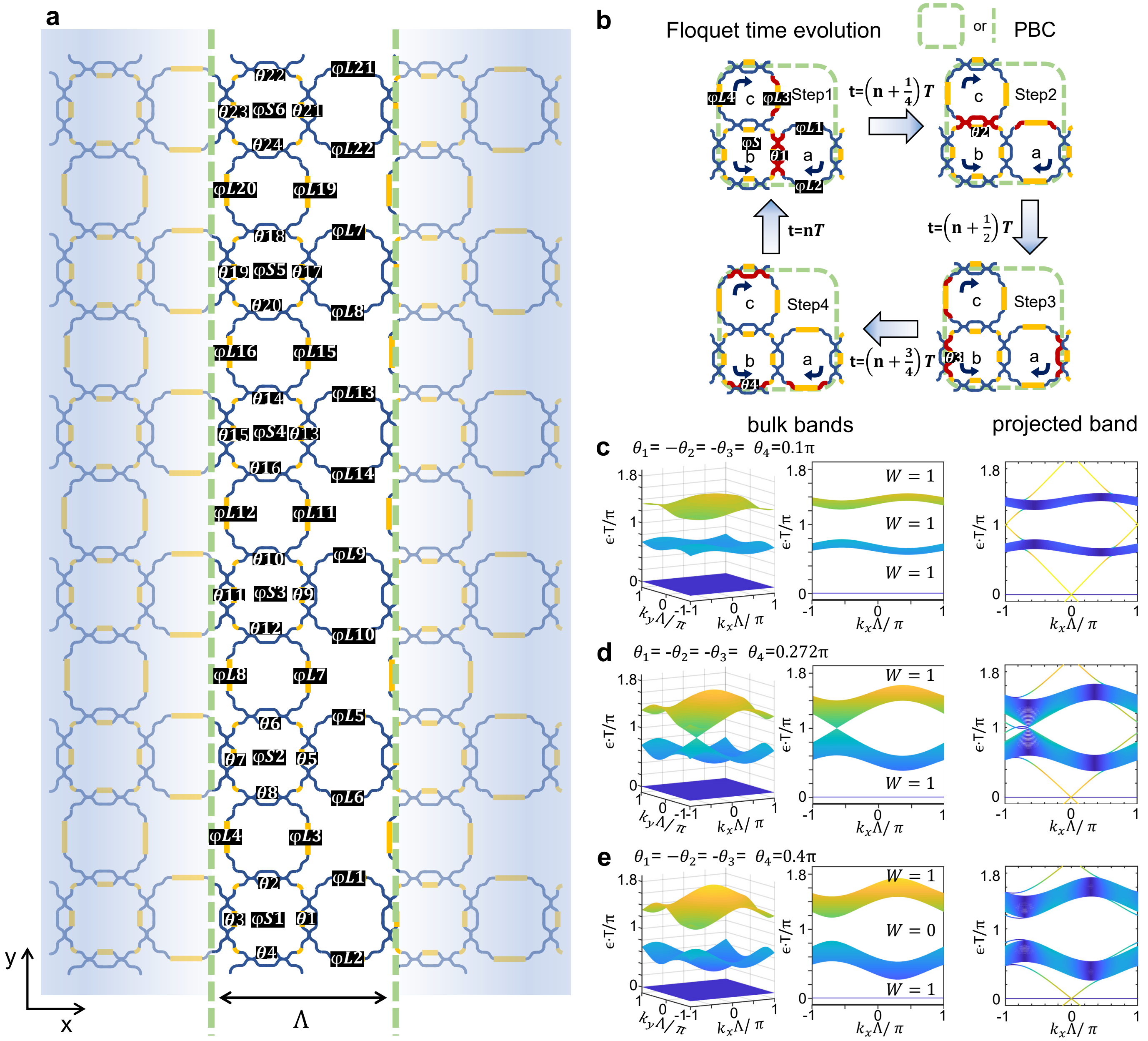

Fig. S17 Microring-based Floquet band theory. **a**, A typical supercell with 6 units in the y direction but periodically infinite in the x direction. All 52 tunable parameters in the 6×1 supercell are labelled. Topological edge modes and projected bands can be reflected because of the existance of boundaries. **b**, Four-step Floquet time evolution in a three-particle Bloch bulk cell. Note that the periodic time evolution in a 3-particle unit cell (containing 1 site rings and 2 link rings) are equally divided into 4 steps with the same evolution time. To ensure the uniformity of perimeter of all rings (i.e, the same evolution period T in the Floquet model), the length of the connection link (the connecting waveguides in link rings) is exactly chosen as quarter of the perimeter. **c-e**, Bulk bands (left panels), bulk bands in k_y direction and projected band (right panels) at different stages in a θ-TPT. The bands are plotted in a shifted window of [-0.1π,1.9π] to show a narrow bulk band at ϵ = 0.

where Λ is the period in real space, kx and ky are transverse momentum in the x and y directions, respectively. Then an effective Floquet Hamiltonian $H_{eff}(k)$ is obtained

$$H_{eff} = \frac{i}{T} \ln U_F. \tag{S5}$$

Time-ordering plays a key role in Floquet modulation, which makes H_{eff} significantly different from a simple temporal average. For a given set of parameters, the band structure can be obtained by solving the eigen-energy. The left panels in Figs. S17c-e demonstrate calculated bands in PBC in another θ-TPT. According to the bulk-boundary correspondence, their topology in OBC can be accurately predicted by topological invariants. For this system, the topological invariant winding number ($\mathcal{W}_\epsilon$) describes the topology of bandgaps. $\mathcal{W}_\epsilon$ describes the topology of an open bandgap at quasi-energy ϵ, $\mathcal{W}_\epsilon = 1$ for non-trivial bandgaps and $\mathcal{W}_\epsilon = 0$ for trivial bandgaps. It can be calculated as:

$$\mathcal{W}_\epsilon = \frac{1}{8\pi^2} \int_0^T dt \int dk_x dk_y \mathrm{Tr}(U_\epsilon^{-1} \partial_t U_\epsilon [U_\epsilon^{-1} \partial_{k_x} U_\epsilon, U_\epsilon^{-1} \partial_{k_y} U_\epsilon]), \tag{S6}$$

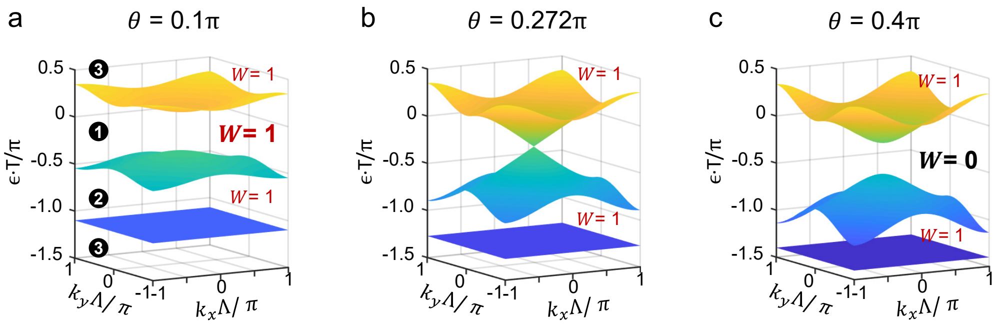

Fig. S18 Evolution of bulk bands in θ-TPTs. Evolution of bulk bands in θ-TPTs in the main text. When θ increases, bandgap ❶ close and reopen at $\theta = 0.272\pi$, accompanied with disappearance of topological edge modes and changes on topological invariants.

where U_ϵ is U_F with a continuous deformation to keep the bandgap at ϵ open. Winding numbers at different bandgaps are expressed in related positions in the middle panels of Figs. S17c-e, which display the bulk bands from a perspective perpendicular to the $\epsilon - k_x$ plane. Obviously, there is no topological edge mode in an infinitely repeated bulk cell. To intuitively distinguish the topology, projected bands are simulated by calculated a supercell. As shown in Fig.S17a, a supercell finite in y direction and infinite in x direction is considered. The bulk bands in projected bands in the right panels of Figs. S17c-e are the same as the results in the middle. Significantly, the emergence of topological edge modes fully obey the predictions of topological invariants. The evolution of bulk bands in θ-TPTs in Fig.2 are complemented in Fig.S18.

Supplementary Note 4: Observations of CROW chain, SSH model and non-Hermitian skin effect in 1D lattices

The well-known and widely used 1D models are implemented on a 2D array, also allowing for operations that are difficult to achieve in 1D. Three representative models: 1D-CROW chain, SSH model and non-Hermitian skin effect, are presented to show the diversity of lattices that can be simulated on the topological chip.

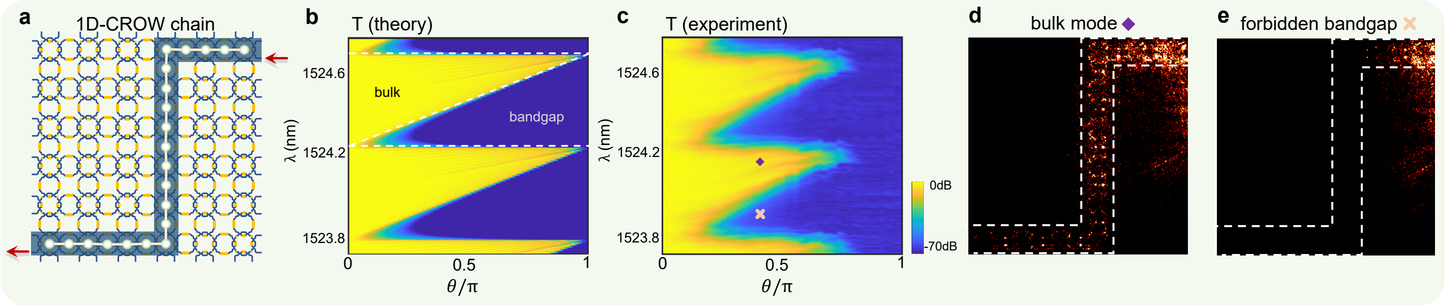

Fig. S19 CROW chains in a 1D lattice. **a**, Schematic of a 1D-CROW chain. All microrings and their coupling are programmed to be identical. Following the optical I/O ports for excitation and detection in (**a**), 2D-transmission spectra in wavelength λ and the parameter θ on coupling strength are (**b**) theoretically calculated and (**c**) experimentally measured, respectively. Real-space distributions of electromagnetic field of (**d**) bulk modes and (**e**) forbidden bandgaps are measured, corresponding to the marks in (**c**).

CROW chains are one of the most widely used optical structures in integrated optics as filters, wavelength division multiplexer and optical delaylines. Multiple identical microrings cascaded together can achieve a flatter high-transmission window and deeper filtering depth compared to a single ring. From a perspective of photonic crystals, the band-pass windows correspond to bulk bands and the band-stop windows correspond to forbidden bandgaps. Both of them are sensitive to disorder-induced deformation of bulk bands. Therefore, CROWs are highly susceptible to fabrication disorders, especially in passive integrated chips, which significantly limits their applications. For the programmable integrated topological photonic chip with arbitrary operations in the lattice, it is not difficult to construct an ideal CROW chain. Fig.S19a displays a 21-site 1D-CROW chain and the dark green background highlights the working area on the chip. All microrings in CROW are resonant and their coupling strength are identically quantified by θ as other models in the main text. Theoretical (Fig.S19b) and experimental (Fig.S19c) 2D-transmission spectra in wavelength λ and coupling parameter θ are highly consistent. As a trvial insulator, there is no topological phase transition but the ratio between the bulk band and the bandgap in one FSR decreases in larger θ (weaker coupling). And completely distinct optical fields in bulk modes and forbidden bandgaps are shown in Fig.S19d and Fig.S19e, rspectively. Considering its additional ability to implement a global phase shift, the CROW chain can be used as a tunable filter. Note that the attenuation of light under large θ in experimental 2D spectra is due to the enhancement of the resonant effect, which increases the effective optical path and therefore increases the transmission loss. Besides, The relationship between the parameter θ and the power transmittance of MZIs is not a linear function. The slope of $T_{MZI}(\theta) = \cos^2(\theta/2)$ is small around $\theta = 0$ and $\theta = \pi$. For example, at $\theta = 0.8\pi$, the power transmittance of a MZI has already dropped to 9.5%, which is very close to 0% at $\theta = 1\pi$.

SSH model is one of the most famous topological models. The periodic altering between strong coupling and weak coupling makes the bands protected by chiral symmetry. Topological bound modes appear at the boundaries and defects in weak coupling. A 1D-SSH chain in Fig.S20a is constructed by modulating the coupling strength in a CROW chain, where single-bar represents weak coupling ($\theta = 0.5\pi$)

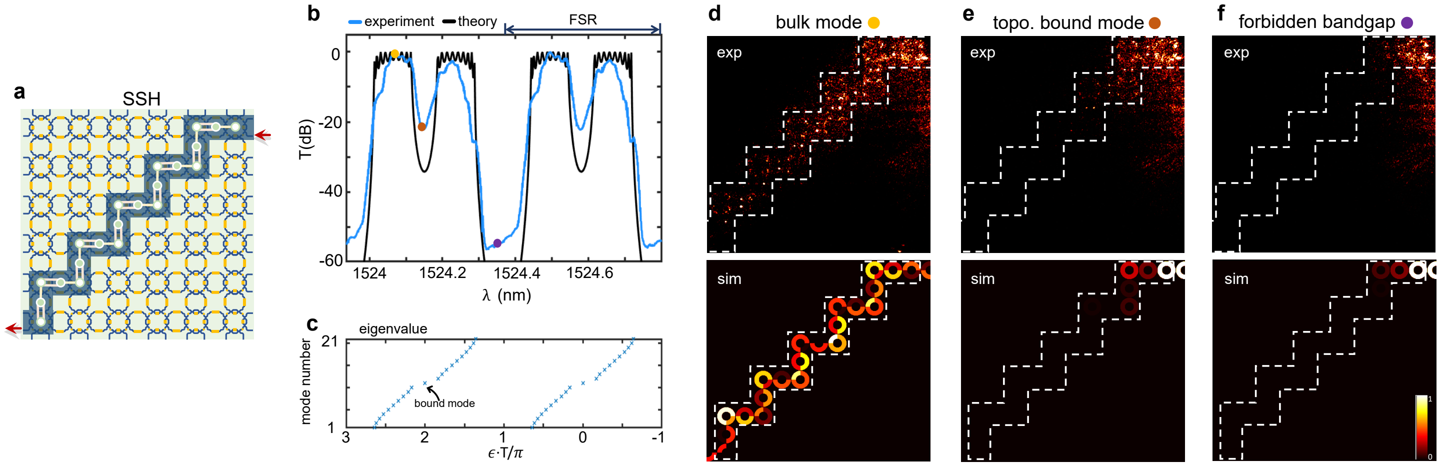

Fig. S20 SSH model in a 1D lattice. **a**, Schematic of SSH model. The coupling strength between neighboring microrings periodically alters between strong coupling (double-bar, $\theta = 0.2\pi$) and weak coupling (single-bar, $\theta = 0.5\pi$). **b** and **c**, theoretical and experimental transmission spectra and corresponding eigenvalue spectra for the 1D-SSH model. Measured (upper panels) and simulated (lower panels) real-space distributions of electromagnetic field of (**d**) bulk modes, (**e**) topological bound modes and (**f**) forbidden bandgaps are associated with the marks in (**b**), respectively.

and double-bar represents strong coupling ($\theta = 0.2\pi$). Theoretical and experimental transmission spectra are plotted in Fig.S20b, together with the corresponding eigenvalue spectra in Fig.S20c. Two bulk bands in a FSR support photon transport as shown in Fig.S20d, but they are intrinsically sensitive to disorders. Topological bound mode is the only mode exists at the bandgap across $\epsilon = 0$. And the distribution of topological bound states is localised at the edge of topological insulators and exponential decay to the bulk sites. While another wider and deeper dip is associated with forbidden bandgaps. The reason for optical blocking here is the absence of any states between the bands. Therefore, light is reflected immediately at the input port. This difference in real space was also observed experimentally and theoretically in Fig.S20e and f.

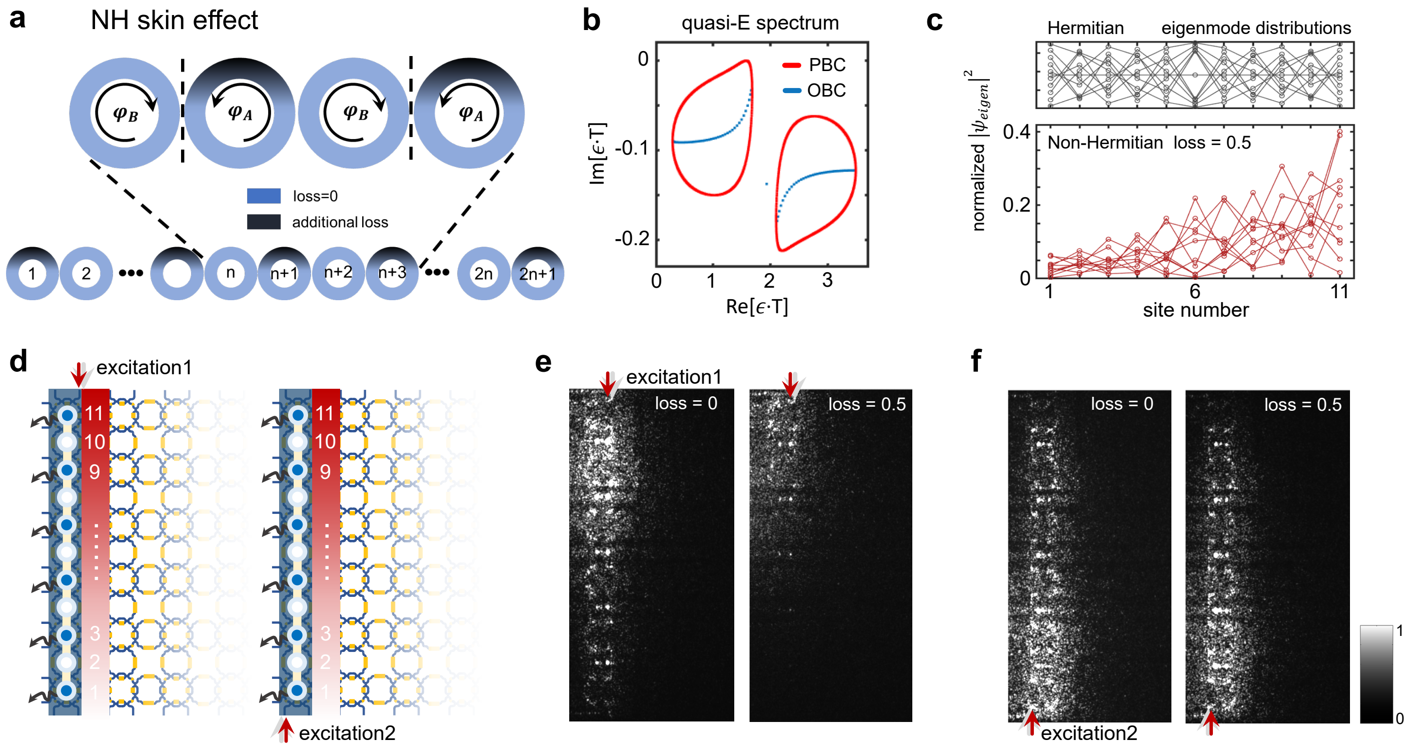

Fig. S21 Non-Hermitian skin effect. **a**, Non-Hermitian models in a Floquet ring chain. A unit cell consists of two coupled microrings in different phases and opposite pseudo-spin. Additional losses are added to the upper paths of ring A. **b**, Theoretical complex quasi-energy spectra in PBC and OBC. The coupling parameter θ between neighboring rings is 0.5π. The phase difference in two coupling rings $\varphi_A - \varphi_B$ is 0.2π. *loss*=0.35, which means 35% power dissipates to the environment after passing the loss regions. 51 rings is considered in OBC. **c**, Distributions of eigenmodes at *loss*=0 and *loss*=0.5 are plotted together. Distribution of the non-Hermitian eigenmodes exhibits a pronounced skewed trend to sites with larger numbers. **d**, Schematic of experimental implementation on the chip. The dissipation to environment is achieved by coupling to the output ports. Excitation from upper and lower ports are implemented, respectively. **e** and **f**, Contrastive infrared imaging of optical field at *loss*=0 and *loss*=0.5 with excitation from upper and lower ports, respectively.

Non-Hermiticity has brought new physics and novel phenomenon to topological band theory in recent years. Non-Hermitian skin effect (NHSE), as an anomalous and unique phenomenon in non-Hermitian system, has attracted significant attention. Despite extensive theoretical studies, precise control of non-Hermitian terms still poses challenges in experimental implementations. Here, we implement a loss-controlled non-Hermitian Floquet model to demonstrate NHSE. As shown in Fig.S21a, two microrings in opposite pseudo-spins couple together forming a unit cell. Two rings A and B holds different phases and the upper paths of ring A introduce additional losses to environment. Experimental design on the topological chip is illustrated in Fig.S21d. 11 microrings at the boundary of device are selected, where a site ring and a link ring consist a unit cell and site rings can introduce additional losses by coupling to the output ports. The additional losses can be quantified by the power transmittance of MZIs. For example, when coupling strength to output port $\theta_{loss} = 0.6\pi$, the loss is quantified as $loss = \cos^2(\theta_{loss}/2) = 0.35$. The coupling parameters θ between neighboring rings are all set to 0.5π. And a 0.2π phase difference between $\varphi_A$andφ_B separates two bulk bands in energy-moment space. With addition losses, the quasi-energy becomes complex. In Fig.S21b, the quasi-energy in periodic boundary condition (PBC) and open boundary condition (OBC) are very distinct. The former consists of two closed loops, while the latter consists of two open lines. For the non-Hermitian system under more realistic OBC, the distribution of the eigenmodes exhibits a pronouncedly skewed trend to sites with larger numbers, compared to the Hermitian case (Fig.S21c). And we experimentally verified the NHSE in Fig.S21e, f. In a Hermitian system, transmissive optical fields are exhibited for both incidences. With additional *loss*, upper-skewed trend obviously appears for upper incidence, while the light still propagates to the upper ports for lower incidence. Besides the non-Hermiticity induced by unbalanced gain-loss, more unique phenomena in non-Hermitian system and their combination with topological insulators can be studied on the programmable integrated photonic topological chip.

Supplementary Note 5: Topological Anderson insulators in a Floquet lattice

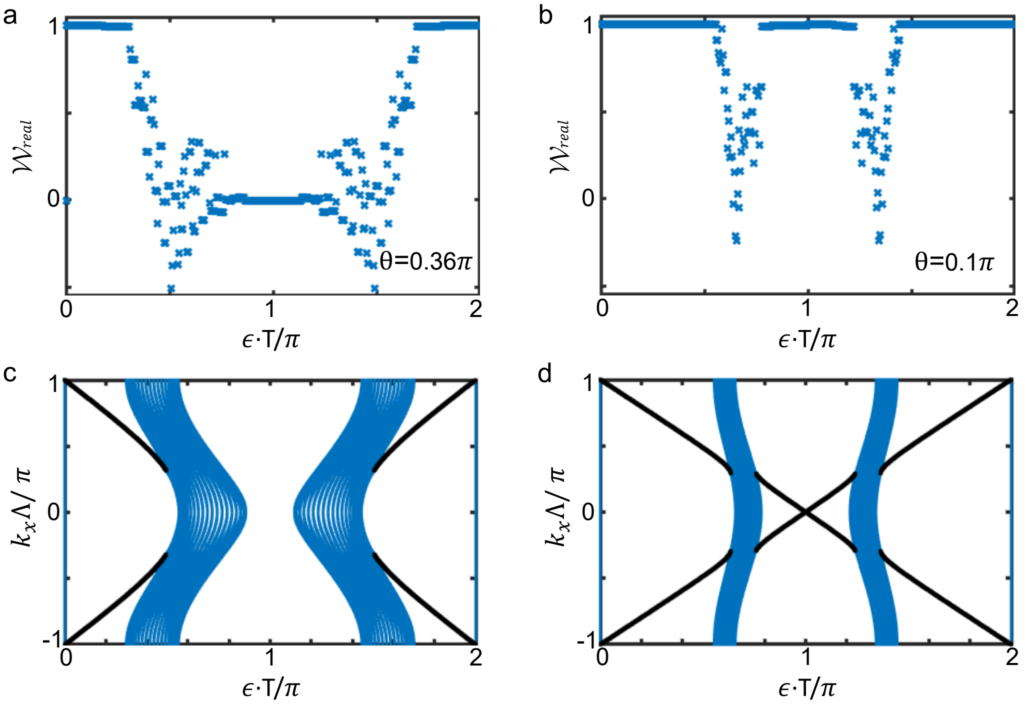

Fig. S22 Real space winding number. Calculated real-space winding number $\mathcal{W}_{real}$ for three-microring models in (**a**) CI phase ($\theta = 0.36\pi$) and (**b**) AFTI phase ($\theta = 0.1\pi$). Corresponding projected bands in (**c**) and (**d**) draw a clear distinction for edge modes, bulk modes and forbidden bandgaps. Similar to the winding number in k space, $\mathcal{W}_{real}$ remains stable around 1 for edge modes and 0 for forbidden bandgaps. While it fluctuates dramatically when ϵ is related to the bulk states, and it does not carry meaningful information.

Topological Anderson insulators are counterintuitive topological phenomena in random media. The conducting states emerge at the forbidden bandgaps of TIs with strong disorders. The statistical results of the TAI boundary modes have been experimentally demonstrated in Fig.4. In this section, theoretical calculation and simulation results are prepared for supplement. Besides, the experiment on a trivial 1D-CROW, which removed all the bulk rings in the FTI, provides a reference for comparison.

The topology of a disordered system cannot be described by common topological invariants as the local disorders destroy the original spatial periodicity. Real-space winding number ($\mathcal{W}_{real}$) is adopted for our Floquet lattice. As a real-space generalization of winding number, real-space winding number is widely used to describe the topology in amorphous topological insulators and fractal topological insulators. And it can be calculated as

$$\mathcal{W}_{real}[U_\epsilon] = -\frac{1}{2}\int_0^T dt Tr\{U_\epsilon^{-1}\partial_t U_\epsilon[U_\epsilon^{-1}[U_\epsilon, X], U_\epsilon^{-1}[U_\epsilon, Y]]\}, \tag{S7}$$

where, X and Y are the position operators in x and y direction mapping to real-space cells in bulk, respectively. Similar to the $\mathcal{W}_\epsilon$ in k space, $\mathcal{W}_{real}$ related to non-trivial bandgaps approaches 1, while it fluctuates around 0 for trivial bandgaps. An illustration for finite-sized FTI is shown in Fig.S22.

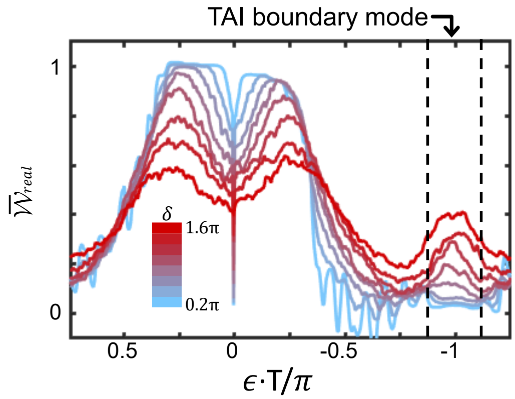

Fig. S23 Real-space winding number in topological Anderson phase transition. Averaged real-space winding number with an increasing δ from 0.2π to 1.6π at an interval of 0.2π. 100 samples at the same δ are considered. The growing peak at the original forbidden bandgaps in $\mathcal{W}_{real}$ indicates the topologcial Anderson phase transition.

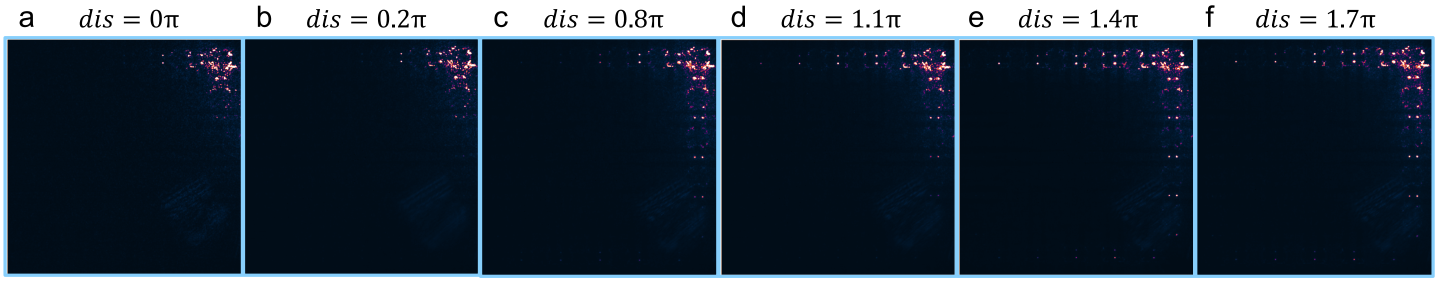

Fig. S24 Infrared imaging of trivial CROW with strong disorders. Accumulation of real-space infrared imaging over 100 samples in the forbidden bandgaps of 1D-CROW with strong phase disorders. Diffusion in x and y directions is observed and quickly decays in both directions, distinct from the unidirectional transport of TAI boundary states.

As a complementary experiment to the trivial CROW under strong disorders in the main text, we also recorded its changes in real space. The statistical real-space distributions over 100 samples in Figs. S24a-f display the diffusive behavior under strong disorder, which is clearly distinct from the unidirectional transport of the Anderson topological boundary states.

Undoubtedly, the programmable and reconfigurable platform for topological research has greatly facilitated the study of statistical properties in topological phenomena. These explorations help us uncover orderly patterns within the realm of disorder. We believe more programmable topological devices will be employed in further investigations.

Supplementary Note 6: Scheme of a programmable topological photonic chip based on a hexagonal lattice

The recirculating photonc circuits, either in hexagonal mesh[53,54] or squared[52] mesh, are different to conventional linear-optical waveguide circuits with only forward operations of classical[43,44,50] and quantum[47,48,51] states of light, while they both possess unique backward (recirculating) operations in the lattice of optical resonators. Such unique capabilities of backward operations allowing coupling of optical microresonators in the lattice is the key to emulate topological materials and physics in our work. In the current topological chip, we use a unit cell consisting of 3 identical rings arranged periodically in space to form a squared lattice. The reason of choosing square-based lattices is that the 3-particle model (see Supplementary Note 3), well fit the structure of squared mesh, allows concise modelling of the Floquet Tis. The Floquet model describes the periodically spatial evolution of light in the lattice, equivalent to evolution under a periodically temporal driven Hamiltonian. The 3-particle model in square mesh possesses a rich phase diagram and can be realized on the programmable photonic chip with sufficient scales. Without breaking time-reversal symmetry, the control of the temporal sequence of inter-ring couplings generates topological boundary states relying on the pseudo-spin direction within the ring.

However, both of the hexagonal and squared meshes can be adopted as the generic topological photonic platform. As we have already shown in Fig.5 in main text, the squared mesh-based topological insulators can be translated to hexagonal mesh-based topological insulators, by reconfiguring the chip. These topological insulators can also be achieved on a programmable hexagonal mesh[53,54]. In analogue to natural materials that have different types of lattices, programmable photonic chips can also have different architectures. When the hexagonal mesh has sufficient tunable components, it can play as a programmable topological chip. Figure S25a shows an alternative scheme of topological insulators based on the hexagonal mesh, with 6-by-6 unit cells. The topological insulator based on hexagonal mesh consists of 121 microrings and 270 MZIs tuning by 540 independent phase shifters (more than those in the squared mesh). Note that the Floquet models cannot be directly implemented in the dense arranged hexagonal lattice in Fig.S25a, because the temporal requirement of Floquet models cannot be met. For example, one loop in a hexagonal ring needs 6 steps. After coupling to two adjacent ring and then coupling back, light finally returns to the starting position, but the time span may not be a multiple of 6 steps. It may even flip the original direction of quasi-spin in the ring. However, as long as the tunablity of the programmable photonic chip is sufficient enough, it can play as topological insulators. In

Fig.S25b, an equivalent Floquet topological insulator in a 6-by-6 unit cells lattice is sketched. The MZIs that connect two link rings have to be set to bar states, and part of rings are isolated to the topological insulators by tuning their edges to bar states.

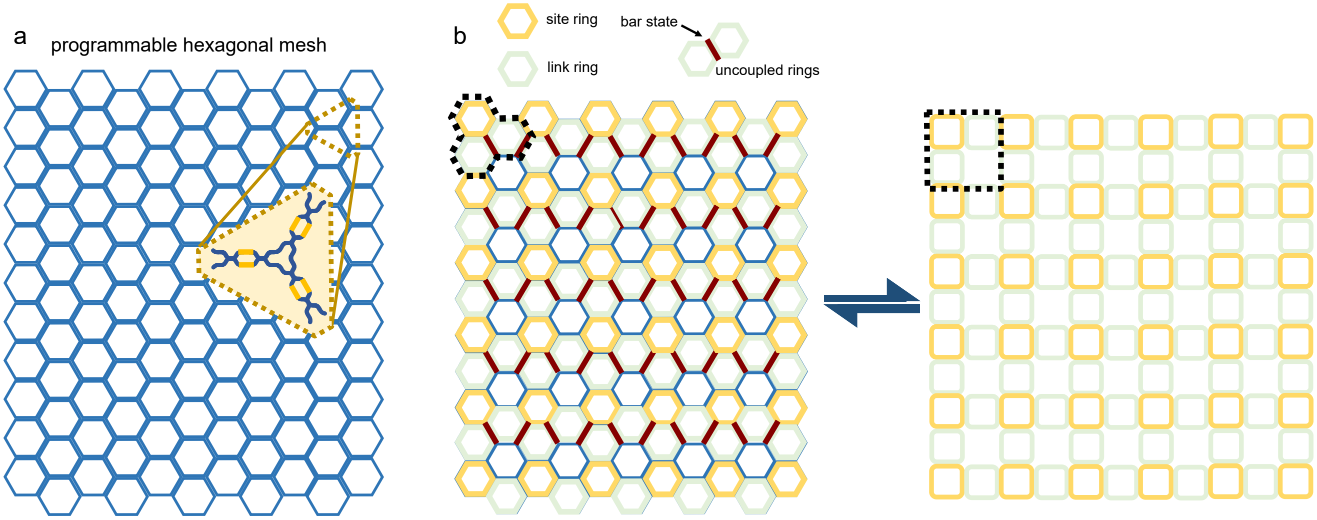

Fig. S25 A scheme for programmable topological photonic chip based on a hexagonal mesh. a, Schematic of a programmable photonic chip in a hexagonal mesh, which was proposed in references [53, 54]. It consists of 121 microrings that are densely arranged and coupled by 270 MZIs (540 independent phase shifters). In this scheme, two tunable phase shifters are designed on both two paths in a MZI, respectively. The coupling strength can be manipulated by the phase difference between two paths, and the propagation phase can be adjusted by simultaneously increasing or decreasing the phases on the two paths. **b**, An equivalent Floquet topological lattice in a hexagonal mesh. The site rings and link rings are indicated in yellow and green, respectively. Those adjacent link rings are not coupled. That being said, the MZIs that connect two link rings have to be set to the bar states. Part of rings are isolated to the topological insulators by tuning their edges to the bar states. The equivalent Floquet topological insulator is also in a 6 unit cells by 6 unit cells lattices as the one in the main text. A 3-particle unit cell is enhanced by dashed lines.

Supplementary Note 7: The scalability of programmable topological photonic chip

The emergence of topological phases relies on Floquet type periodic evolution of light in the recruiting lattice. In our current experiment, a square lattice of six-by-six unit cells consisting of 96 microrings is devised. The topological chip monolithically integrates 2712 components in a 11mm $\times$ 7mm footprint, including 408 low-loss directional couplers, 300 thermo-optic phase shifters with 528 thermal isolators (allowing 30mW low-power consumption), 48 grating couplers for optical access and 120 tapping ports for light field imaging, 600 electronic access and 708 transmission lines. The realization of this a large-scale programmable photonic device becomes possible, because of the recent rapid development of CMOS-compatible fabrication of silicon photonic circuits. The 248nm deep ultraviolet photolithography was used to define all patterns. The device was fabricated on a 200mm silicon-on-insulator wafer. When further scaling up the topological chip using the current fabrication and control technologies, a smart design of integrated photonic waveguide circuits and electrical routing circuits is necessary. For a programmable topological photonic chip with a N-unit-cell by N-unit-cell (totally $N \times (3N-2)$ mirorings), the number of tunable phase shifters is proportional to N^2, while the perimeter of the whole microring lattice is only proportional to N. For every tunable phase shifter, its electronic transmission lines are individually routed towards the edge of the chip for electronic wire bonding. Typically, transmission lines are made of 800nm-thick AlCu with a 5um-width and 5um-gaps. Surely, sharing common grounds could further reduce the footprint of electrical routing circuits. That being said, the current approach is limited by electrical layer routing, instead of photonic layer design (only occupying a footprint of 4mm $\times$ 4mm, while the footprint for optical + electronic layers is 7mm $\times$ 11mm). In a typical writing area of DUV lithography (25mm $\times$ 33mm, or 50mm $\times$ 33mm by stitching two area), one could integrated about 37-by-50 unit cells with 5463 rings or 74-by-50 unit cells with 10967 rings by fied stitching lithography. Importantly, one note that operating such large-scale integrated photonic devices, optical loss, crosstalk and power consumption, and their scalable electronic controls, again have to be taken into account. Advanced silicon-based integrated photonics technologies may provide the ultimate solutions for very-large-scale programable topological insulators, such as heterogeneous integration and electro-optical packaging /co-integration.
